# Mechanical Environment Afforded by Engineered Hydrogel Critically Regulates Survival of Neural Stem Cells Transplanted in the Injured Spinal Cord via Piezo1‐Mediated Mechanotransduction

**DOI:** 10.1002/advs.202507160

**Published:** 2025-11-03

**Authors:** Hee Hwan Park, Yurim Kim, Byeong Seong Jang, Simay Genişcan, Dong Hoon Hwang, Yeojin Seo, Seung‐Ah Jee, Hyo Gyeong Seo, Hyung Soon Kim, Ariandokht Einisadr, Ho‐Jeong Kim, Seolhee Lee, Sangwoo Kwon, Kyung Sook Kim, Kang In Lee, Jae Young Lee, Joo Min Park, Young‐Min Kim, Soo‐Chang Song, Byung Gon Kim

**Affiliations:** ^1^ Department of Brain Science Ajou University School of Medicine Suwon 16499 Republic of Korea; ^2^ Neuroscience Graduate Program, Department of Biomedical Sciences Ajou University Graduate School of Medicine Suwon 16499 Republic of Korea; ^3^ Center for Biomaterials Korea Institute of Science and Technology Seoul 02792 Republic of Korea; ^4^ University of Science and Technology (UST) Daejeon 34113 Republic of Korea; ^5^ Wonju Medical Industry Technovalley Wonju 26354 Republic of Korea; ^6^ Center for Cognition and Sociality Institute for Basic Science Daejeon 34126 Republic of Korea; ^7^ Department of Biomedical Engineering Ulsan National Institute of Science and Technology (UNIST) Ulsan 44919 Republic of Korea; ^8^ Department of Biomedical Engineering, College of Medicine Kyung Hee University Seoul 130‐710, 02447 Republic of Korea; ^9^ ToolGen Inc. Seoul 07789 Republic of Korea; ^10^ Department of Anatomy Ajou University School of Medicine Suwon 16499 Republic of Korea; ^11^ Department of Neurology Ajou University School of Medicine Suwon 16499 Republic of Korea

**Keywords:** hydrogel, mechanical stiffness, neural stem cell transplantation, Piezo‐1, spinal cord repair

## Abstract

Neural stem cell (NSC) transplantation is a promising therapeutic approach for spinal cord repair, but poor graft survival remains a critical challenge. This work reports that the mechanical properties of the transplantation environment play a crucial role in NSC survival in the injured spinal cord. While this previously developed engineered hydrogel effectively creates extracellular matrix preventing cystic cavity formation, NSCs transplanted as a complex with 10% hydrogel exhibits poor survival. Remarkably, increasing the hydrogel concentration to 16%, creating a fivefold stiffer matrix, significantly enhances NSC graft survival. Using in vitro models with controlled substrate stiffness, this work finds that NSCs on stiffer substrates display enhanced adhesion, complex morphology, and increased viability. Electrophysiological recordings in NSCs reveal pressure‐induced inward currents that are significantly reduced by Piezo1 inhibition. Pharmacological or siRNA inhibition of Piezo1 alters NSC morphology and reduces adhesion specifically on stiffer substrates. Importantly, CRISPR/Cas9‐mediated *Piezo1* gene editing significantly reduces graft survival in vivo when transplanted with 16% hydrogel, confirming that Piezo1‐mediated mechanotransduction is essential for stiffness‐dependent NSC survival. These findings reveal a previously unrecognized mechanism governing graft survival and suggest that optimizing mechanical properties of biomaterial scaffolds or directly targeting Piezo1‐dependent mechanotransduction could substantially improve outcomes of cell‐based therapies for neurological disorders.

## Graphical abstract

Neural stem cell transplantation shows promise for spinal cord repair, but poor graft survival limits therapeutic efficacy. This study demonstrates that increasing hydrogel stiffness from 10% to 16% concentration significantly enhances transplanted neural stem cell survival through Piezo1‐mediated mechanotransduction, revealing mechanical properties as critical determinants of cell‐based therapy success.

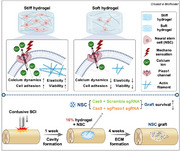

## 1 Introduction

Cell‐based therapy holds significant promise for treating spinal cord injury (SCI), a devastating condition that often leads to permanent neurological deficits.^[^ ^1^ *^,^* ^2^ ^]^ Several mechanisms facilitate the repair of the injured spinal cord, providing neuroprotection by modulating inflammatory responses, altering the regeneration‐inhibiting environment at the injury site, and promoting remyelination in the spared white matter.^[^ ^1^ ^]^ In particular, neural stem or progenitor cells (NSCs) transplanted into the injured spinal cord can potentially differentiate into neurons and glial cells, replace lost tissue, provide neurotrophic support, and may help rebuild neural connections to create a new relay circuit.^[^ ^3^ *^,^* ^4^ ^]^ However, a significant challenge that limits the efficacy of NSC transplantation is poor graft survival, with the majority of transplanted cells dying within days and weeks after transplantation.^[^ ^5^, ^6^, ^7^, ^8^, ^9^ ^]^ The issue of grafted cell survival is not limited to NSCs or cell transplantation for SCI; similar survival constraints have been observed with other cell types, including Schwann cells^[^ ^10^ ^]^ and mesenchymal stem cells,^[^ ^11^ *^,^* ^12^ ^]^ as well as in the context of transplantation for other neurological disorders.^[^ ^13^ *^,^* ^14^ ^]^

Several factors contribute to the hostile microenvironment in injured spinal cord tissue that compromises graft survival, including inflammation, oxidative stress, and lack of appropriate extracellular matrix (ECM) support.^[^ ^7^ *^,^* ^15^ ^]^ To address these challenges, biomaterial scaffolds have been developed to provide structural support and a protective environment for transplanted cells.^[^ ^16^ *^,^* ^17^ ^]^ Among these, hydrogels have emerged as particularly promising biomaterials due to their tunable properties, injectability, and ability to create a permissive environment for cell survival and integration.^[^ ^18^, ^19^, ^20^ ^]^ The physical properties of hydrogels, particularly their mechanical stiffness, significantly influence cellular behaviors such as adhesion, migration, proliferation, and differentiation.^[^ ^21^, ^22^, ^23^ ^]^ However, the impact of hydrogel stiffness on NSC survival after transplantation into the injured spinal cord has not been explored.

We previously engineered a polymer hydrogel composed of imidazole‐poly(organophosphazene) (I‐5 hydrogel) and demonstrated that the imidazole‐mediated interaction with macrophages induces endogenous ECM remodeling at the lesion epicenter, preventing the formation of cystic cavities or tissue defects following contusive SCI.^[^ ^20^ ^]^ While this I‐5 hydrogel effectively created protein‐rich matrices at the lesion epicenter, we observed that NSCs transplanted in combination with this hydrogel still exhibited poor survival. This unexpected finding led us to investigate whether modulating the mechanical properties of the hydrogel could enhance NSC survival after transplantation. In this study, we hypothesized that increasing the stiffness of the I‐5 hydrogel would improve the survival of transplanted NSCs in the injured spinal cord. Furthermore, we sought to identify the cellular and molecular mechanisms underlying the stiffness‐dependent survival of NSCs, focusing on the potential role of Piezo1‐mediated mechanotransduction. Our findings demonstrate a crucial relationship between mechanical cues, Piezo1 activation, and NSC survival, offering new insights for enhancing cell‐based therapies for SCI and other neurological disorders.

## 2 Results

### 2.1 Transplantation of NSCs Complexed with I‐5 Hydrogel Does Not Improve Graft Survival

**Figure 1:**
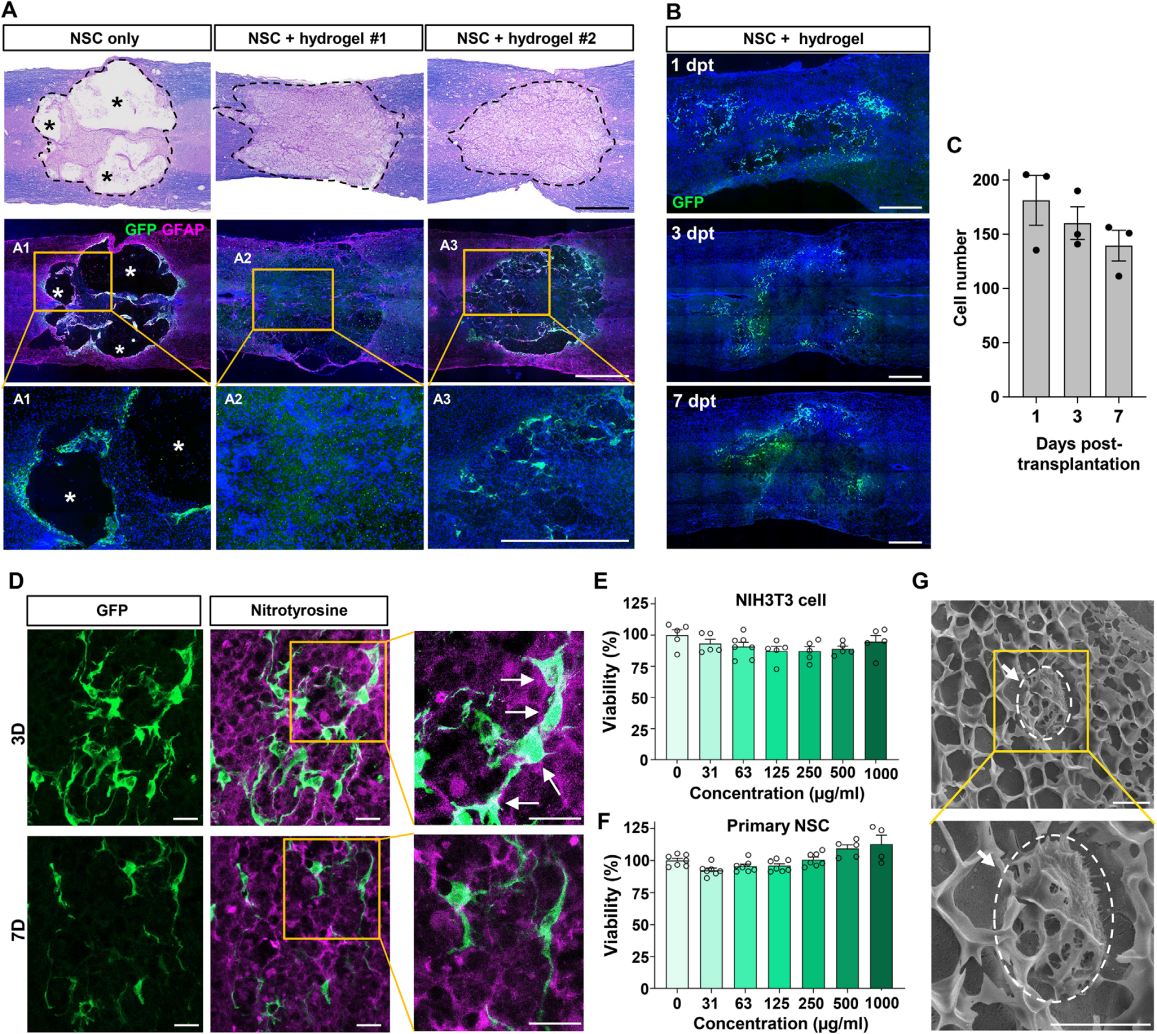
Transplantation of NSCs with hydrogel does not improve graft survival. A) Representative images of longitudinal spinal cord sections obtained from animals transplanted with NSC grafts with or without hydrogel at 4 weeks after transplantation (5 weeks after injury). NSCs obtained from green fluorescent protein (GFP)‐expressing SD rats were injected into the spinal cord at 7 days after contusive injury at T9 level. When NSCs were transplanted without hydrogel (left), the injection was done twice at 2 mm rostral and caudal to the epicenter. For transplantation with hydrogel, injection was done once at the epicenter. Huge cystic cavities were observed when NSCs were injected without hydrogel, and only very few GFP‐positive NSCs were observed along the cavity walls (left). In contrast, injection of NSCs with hydrogel resulted in the formation of ECM at the lesion center. However, there was no visible GFP‐positive NSCs (middle, NSC + hydrogel #1) or very few NSCs surviving within the newly created ECM (right, NSC + hydrogel #1). Boxed regions in the middle panel are magnified below (A1‐A3). Asterisk indicates cystic cavity at the lesion epicenter. Scale bars = 500 µm. B) Representative images of longitudinal spinal cord sections from animals with NSC grafts and hydrogel, taken at 1, 3, and 7 days post‐transplantation (dpts). Scale bars = 500 µm. C) Quantification graph of surviving NSCs at 1, 3, and 7 dpts. Each dot represents an independent animal. Error bars represent SEM. The number of surviving NSCs was already low at 1 day and further decreased at 3 and 7 days, indicating that NSC death occurs very early after transplantation. D) Representative confocal images of surviving NSC grafts at 3 and 7 dpts. Immunostaining with anti‐nitrotyrosine antibodies was employed to visualize the nitrated proteins formed by reactive peroxynitrite molecules. White arrows in the magnified images indicate co‐localization of NSC grafts with nitrated proteins. Scale bars = 20 µm. E,F) Cellular cytotoxicity assay of hydrogel solution, imidazole‐poly(polyorganophosphazene) (I‐5). There was no toxicity of the hydrogel solution at up to 1 mg mL^−1^ concentrations for both NIH3T3 cells (E) and primary NSCs (F). Error bars represent SEM. G) Representative cryogenic scanning EM image of I‐5 hydrogel complexed with NSCs. The size of pores formed in a gel state was smaller than that of NSCs. The white arrows indicate an NSC attached to the hydrogel. Scale bar = 5 µm.

Our previous study reported that NSCs transplanted into the spinal cord after a contusion injury exhibit poor survival.^[^ ^6^ ^]^ Consistent with this finding, the majority of NSCs grafted 1 week after the contusion injury disappeared within 4 weeks, leaving only a few surviving cells along the cavity walls (**Figure** **1**A). At the lesion epicenter, we consistently observed the formation of cystic cavities of varying sizes and geometries, suggesting that the absence of ECM at the core of the lesion may contribute to their poor survival.

We predicted that when NSCs are combined with I‐5 hydrogel, which has been shown to prevent cavity formation by remodeling the ECM,^[^ ^20^ ^]^ the chances of NSC survival would improve because the hydrogel can create a protein‐based matrix that allows NSCs to adhere to and interact with their surrounding matrix environment. As expected, transplantation of NSCs combined with I‐5 hydrogel resulted in the formation of eosin‐positive matrices at the lesion core, where in the absence of the hydrogel, we would have seen tissue defects accompanied by cystic spaces. However, contrary to our expectations, there were very few NSCs (see Figure 1A, case #1) or only a small number of NSCs present in the newly created ECM (see Figure 1A, case #2). Our previous study indicated that significant loss of grafted NSCs occurs as early as 3 days post‐transplantation.^[^ ^6^ ^]^ We noticed that the number of surviving NSCs had already decreased 1 day post‐transplantation (dpt), and the NSC survival was further reduced at 3 and 7 dpts (Figure 1B,C), suggesting that NSCs undergo substantial death within several days following transplantation, when I‐5 hydrogel has not yet fully degraded and active ECM remodeling is still ongoing.^[^ ^20^ ^]^ One possible mechanism mediating the death of grafted NSCs in the injured spinal cord involves reactive nitrogen species.^[^ ^6^ *^,^* ^24^ ^]^ Nitrotyrosine antibodies were used to visualize the nitration of tissue proteins by reactive peroxynitrite molecules.^[^ ^25^ ^]^ At 3 dpt, we frequently observed that the GFP‐positive NSCs colocalized with nitrotyrosine immunoreactivity (Figure 1D). However, by 7 days, the proportion of nitrotyrosine‐positive NSCs appeared to decrease (Figure 1D), indicating that the NSCs were primarily exposed to reactive nitrogen species during the first few days following transplantation.

To exclude any chance of cytotoxicity from the I‐5 polymer molecules, we conducted a cell survival assay. In this assay, cells were treated with different concentrations of the I‐5 polymer solution. Either the NIH3T3 fibroblast cell line or primary NSCs showed no evidence of cytotoxicity at concentrations up to 1000 µg mL^−1^ (Figure 1E,F). It is also conceivable that the size of NSCs is smaller than the pores formed within the hydrogel, which could lead to inadequate encapsulation by the hydrogel. To investigate this possibility, a 10% I‐5 hydrogel complexed with NSCs was subjected to cryogenic SEM to compare the sizes of the NSCs and the hydrogel pores (Figure 1G). The diameter of the pores in the hydrogel ranged from one to two micrometers to approximately five micrometers, with very few pores exceeding five micrometers in diameter. In contrast, the majority of NSCs growing within the hydrogel had diameters larger than five micrometers. This indicates that grafted NSCs are unlikely to be filtered out through the hydrogel pores, reducing the likelihood that they were directly exposed to a hostile injury microenvironment. Some studies have reported that when transplanted cells pass through a syringe needle, their cellular membranes may be damaged due to shear stress, which is proportional to the viscosity of the cell suspension.^[^ ^7^ *^,^* ^26^ ^]^ We speculated that NSCs complexed with viscous I‐5 hydrogel might experience significant shear stress during the injection process. To investigate this, we examined whether there was an increase in the proportion of dead NSCs after the cells in the I‐5 polymer complex underwent the injection procedure using the same apparatus and techniques. However, our results indicated that there was no change in the number of dead NSCs before and after the injection (Figure S1, Supporting Information). We also investigated whether the survival of grafted NSCs could be enhanced by incorporating growth factors into the hydrogel by activating survival pathways in NSCs. Our previous study demonstrated that insulin‐like growth factor‐1 (IGF‐1) signaling is crucial for the survival of NSC grafts.^[^ ^27^ ^]^ However, we did not observe significant improvement in survival when NSCs were combined with hydrogel containing IGF‐1 (Figure S2A, Supporting Information). ECM proteins that promote cellular adhesion are commonly utilized to improve graft survival when engineered hydrogels are employed to support therapeutic cell transplantation.^[^ ^26^ *^,^* ^28^ ^]^ We attempted to incorporate laminin, an ECM protein frequently used to enhance cell attachment, but we failed to increase graft survival (Figure S2B, Supporting Information).

### 2.2 Concentration of Hydrogel Determines the Mechanical Stiffness and Influences the Survival of NSC Grafts in the Injured Spinal Cord

**Figure 2:**
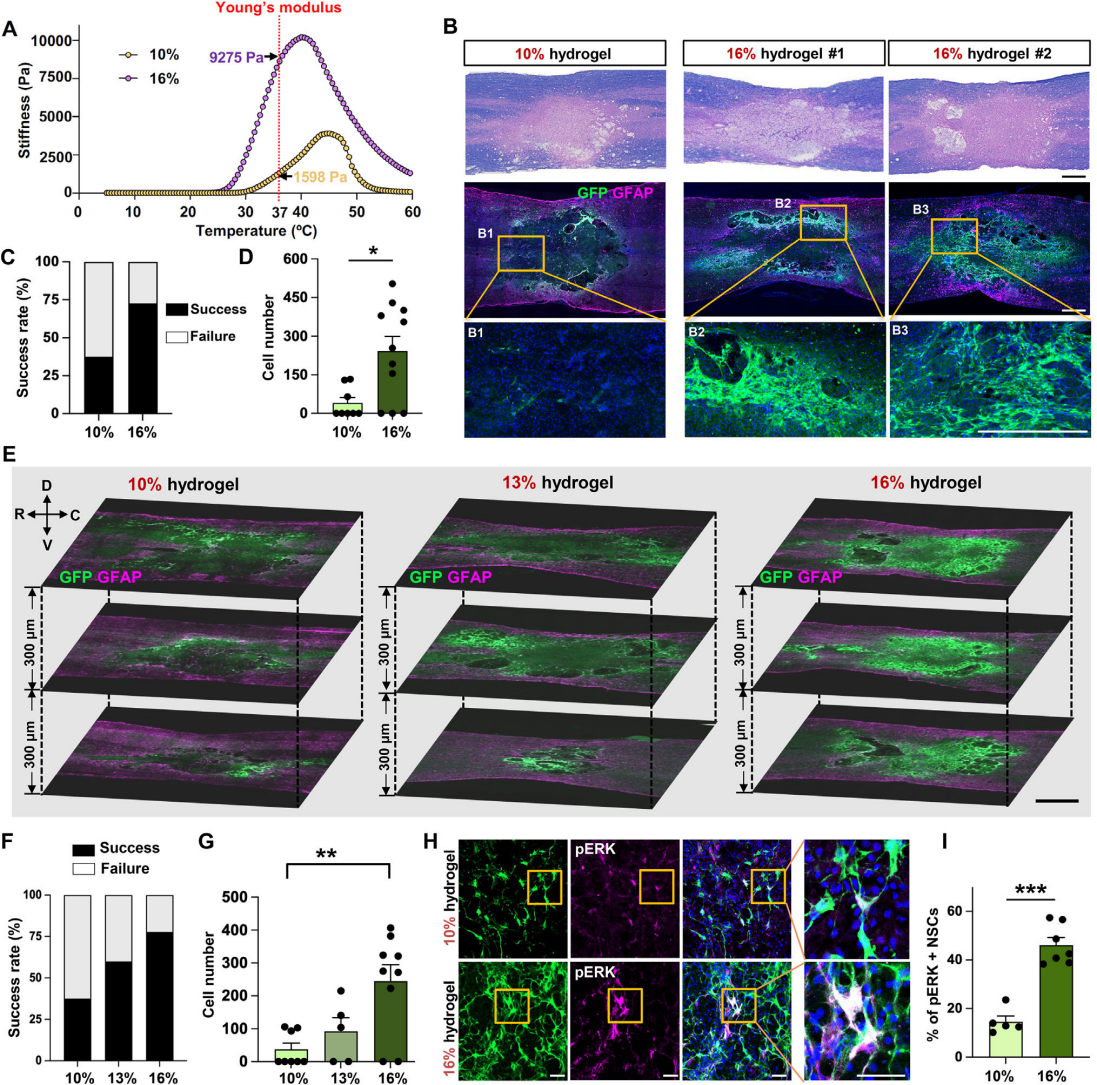
Concentration of hydrogel determines the mechanical stiffness and influences the survival of NSC grafts in the injured spinal cord. A) Stiffness of 10% and 16% I‐5 hydrogel was measured using a rheometer over a temperature range of 5 to 60 °C. B) Spinal cord lesion areas were visualized using eriochrome cyanine and eosin staining (top panel). Surviving NSCs were identified as GFP‐positive cells (green, middle panel), and lesion borders were demarcated by GFAP immunostaining (magenta, middle panel). Enhanced graft survival was noted when NSCs were transplanted with 16% hydrogel (two representative images on the right) compared to the 10% hydrogel (left). Boxed regions in the middle panel are magnified below (B1‐B3). Scale bar = 500 µm. C,D) Quantitative graphs comparing the rate of graft success (C) and the number of GFP‐positive NSCs (D). Graft success was defined as the presence of noticeable GFP positive NSC grafts in at least one tissue section. Error bars represent SEM. * indicates *p* < 0.05 by unpaired *t*‐test. N = 8 and 11 for 10% and 16% hydrogel groups, respectively. E) Representative images of the three longitudinal spinal cord sections containing the lesion epicenter are displayed with a 300 µm interval along the dorsal‐ventral (D‐V) axis. R indicates rostral and C caudal direction. Animals were transplanted with GFP‐positive NSCs with 10%, 13%, or 16% hydrogel after 1 week post‐injury. Samples were collected 4 weeks post‐transplantation (5 weeks post‐injury). Surviving NSCs were identified as GFP‐positive cells (green), and lesion borders were marked by GFAP immunostaining (magenta). Scale bar = 1000 µm. F,G) Quantitative analysis of the graft success rate (F) and the number of GFP‐positive NSCs (G). ** indicates *p* < 0.01 by one‐way ANOVA followed by Tukey's *post hoc* analysis. Error bars represent SEM. N = 8, 5, and 9 animals for 10%, 13, and 16% hydrogel groups, respectively. H) Representative images of phospho‐ERK immunostaining with surviving NSC grafts. Boxed regions are magnified on the right side. Scale bar = 50 µm. I) Quantitative graph showing the proportion of GFP and phospho‐ERK double‐positive cells. Error bars represent SEM. *** indicates *p* < 0.001 by unpaired *t*‐test. N = 5 for 10% and N = 7 animals for 16% hydrogel group.

There is mounting evidence that the physical properties of biological molecules exert crucial roles in determining cellular behaviors.^[^ ^29^ *^–^* ^31^ ^]^ For example, matrix stiffness can modulate cancer cell metastasis, intravasation, and metabolic pathway.^[^ ^32^ *^–^* ^34^ ^]^ Moreover, mechanical cues influence NSC behavior based on substrate stiffness.^[^ ^35^, ^36^, ^37^ ^]^ Recent studies emphasize the significance of the physical stiffness of engineered substrates that utilize artificial biomaterial scaffolds.^[^ ^21^ ^]^ Therefore, we hypothesized that modulating the mechanical stiffness of the I‐5 hydrogel may influence the survival of grafted NSCs transplanted as a complex with hydrogel. Assessment of the mechanical properties of the I‐5 hydrogel using a rheometer indicated that the Young's modulus, which measures the stiffness of solid materials, was found to be 1.6 kPa at 37 °C for 10% I‐5 hydrogel (**Figure** **2**A). This value is markedly lower than the reported value in the rat spinal cord, which is ≈10 kPa.^[^ ^38^ ^]^ Hydrogel stiffness can be controlled by adjusting the polymer concentration.^[^ ^39^ *^–^* ^41^ ^]^ Increasing the hydrogel concentration from 10% to 16% resulted in the stiffness of the 16% hydrogel being approximately five times greater than that of the 10%, reaching 9.3 kPa at 37 °C (Figure 2A). To further characterize the mechanical behavior of I‐5 hydrogels, we performed frequency, strain, and temperature sweep rheological analyses. Both 10% and 16% hydrogels exhibited elastic‐dominant behavior (G′ > G″), maintained linear viscoelasticity up to ≈60% strain, and showed stable structural integrity over time at 37 °C (Figure S3A–C, Supporting Information). Despite differences in stiffness, the two formulations displayed consistent viscoelastic profiles and thermal stability, indicating similar overall rheological behavior. We found that transplanting NSCs complexed with 16% hydrogel led to a significantly improved survival of grafted NSCs compared to transplantation with 10% hydrogel (Figure 2B).

Large clusters of GFP‐positive NSC grafts were frequently located along the periphery of the hydrogel‐created ECM (see Figure 2B, case #1). In some instances, nearly the entire area of the ECM was occupied by NSC grafts (see Figure 2B, case #2). In 11 animals with NSC transplants complexed with 16% hydrogel, 8 (72.7%) animals displayed signs of surviving grafts (Figure 2C). In contrast, NSC grafts were observed in only 3 out of the 8 animals (37.5%) that received transplants with 10% hydrogel (Figure 2C). Additionally, the number of surviving NSCs was approximately six times greater in the group using 16% hydrogel compared to the 10% hydrogel group (Figure 2D).

To confirm that a higher percentage of I‐5 hydrogel supports the survival of NSCs, we designed a new experiment involving a different set of animals. In this experiment, the concentration of the hydrogel was titrated to three different levels; 10%, 13%, and 16%, and NSCs were transplanted as a complex with each of these hydrogel concentrations. The influence of hydrogel concentration on NSC survival was also demonstrated in this replication experiment as the degree of NSC graft survival was correlated with hydrogel concentration (Figure 2E). The proportion of animals with graft success increased with higher hydrogel percentages, with survival rates of 37.5% at 10%, 60.0% at 13%, and 77.8% at 16% (Figure 2F). A one‐way ANOVA revealed a significant difference in the number of surviving NSCs among the three groups with different hydrogel concentrations (*F*_(2, 19)_ = 5.96, *p* < 0.01), and *post hoc* analysis showed a significant difference between the 10% and 16% groups (Figure 2G). We have previously demonstrated that the phosphorylation of ERK, a downstream kinase involved in growth factor‐dependent prosurvival signaling, can serve as a marker for surviving NSCs following transplantation.^[^ ^6^ ^]^ We counted the number of phospho‐ERK (pERK) positive NSCs and found that the proportion of pERK‐positive NSCs was markedly increased in animals transplanted with 16% hydrogel compared to those using 10% hydrogel (Figure 2H,I). Since neuroinflammation at the injury site can significantly impact the survival of transplanted NSCs,^[^ ^42^ ^]^ we investigated whether varying hydrogel concentrations affected the activation of inflammatory cells at the injury site. Consistent with our previous report,^[^ ^20^ ^]^ the intensity of immunoreactivity against Ionized calcium‐binding adaptor molecule 1 (Iba1), a marker for myeloid cell lineage, was notably reduced in animals that received NSC transplantation combined with hydrogel, compared to those that underwent NSC transplantation alone (Figure S4, Supporting Information). However, there were no significant differences in Iba1 immunoreactivity between the groups with different hydrogel concentrations (Figure S4, Supporting Information).

### 2.3 Influence of Hydrogel Concentration on NSC Integration into the Host Spinal Cord

**Figure 3:**
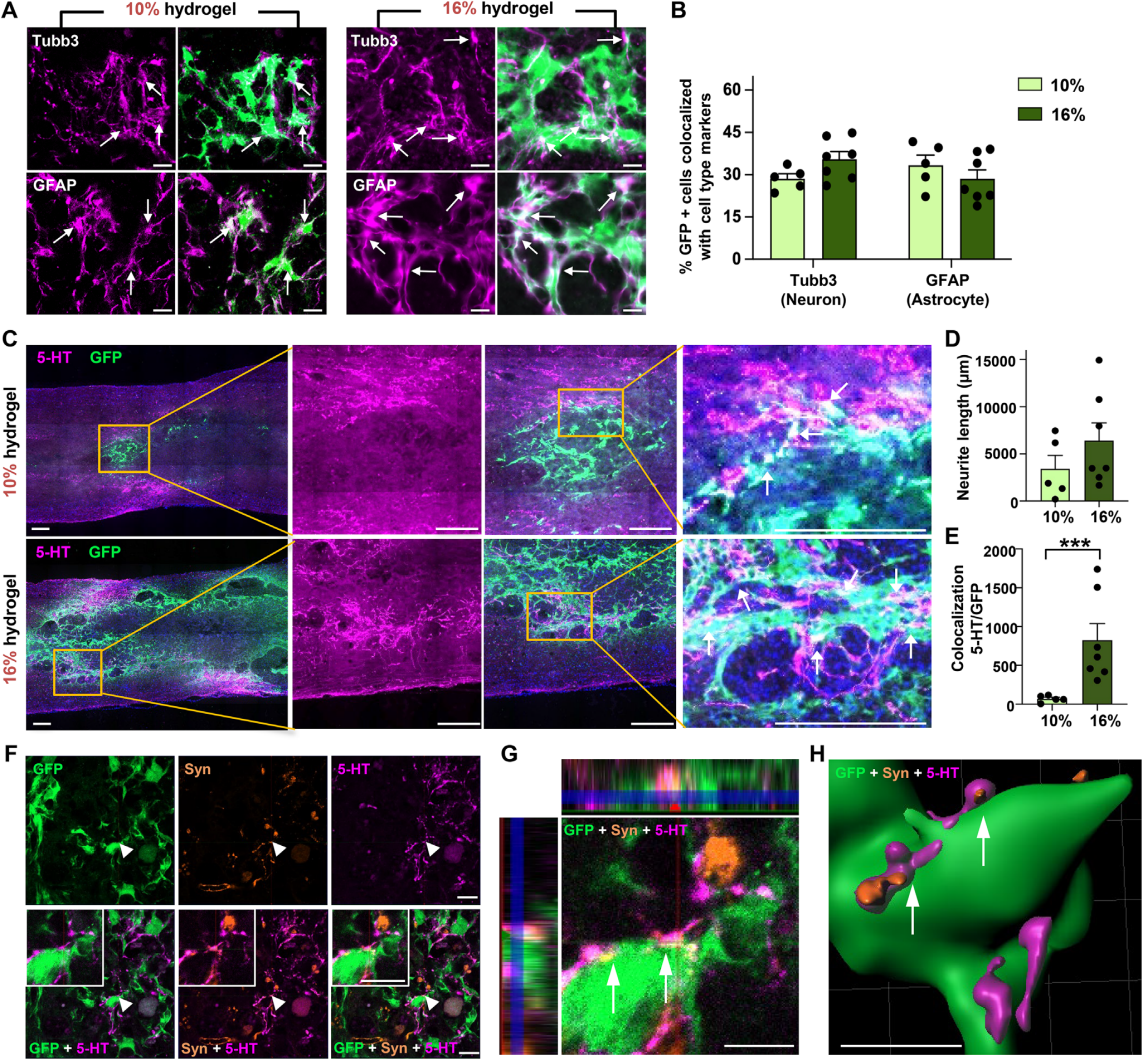
Influence of hydrogel concentration on NSC integration into the host spinal cord. A) Representative images of phenotypic differentiation of NSC grafts into neuronal lineage marked by Tubulin beta 3 (Tubb3) positivity (upper panel) and astrocytic lineage by Glial fibrillary acidic protein (GFAP) positivity (lower panel). White arrows indicate co‐localization of NSC grafts with each cell type marker. Scale bar = 20 µm. B) Quantitative analysis of differentiation percentages in NSC grafts. N = 5 and 7 for 10% and 16% hydrogel groups, respectively. Error bars represent SEM. C) Representative images of longitudinal spinal cord sections immunostained with anti‐5‐HT antibody, serotonergic axon (5‐hydroxytryptamine) fiber marker. Boxed regions are magnified on the right side. White arrows indicate co‐localization of 5‐HT axons (magenta) with NSC grafts (green). Scale bars = 200 µm. D,E) Quantification of 5‐HT+ axon length growing within NSC grafts (D) and co‐localization with NSC grafts (E). *** indicates *p* < 0.001 by unpaired *t*‐test. N = 5 and 7 animals for 10% and 16% groups, respectively. Error bars represent SEM. F) Representative co‐localization images of NSC grafts (GFP, green) with host 5‐HT axons (5‐HT, magenta) and presynaptic vesicle (synaptotagmin, orange). White arrowheads indicate tripartite co‐localization Scale bars = 10 µm. G,H) Orthogonal (G) and 3D‐rendered (H) images showing synaptic integration of NSC grafts with 5‐HT+ presynaptic axons (white arrows). Scale bar = 5 µm.

Previous studies have shown that the mechanical environment in hydrogel scaffolds influences differentiation of stem cells grown on them.^[^ ^43^ *^,^* ^44^ ^]^ Therefore, we assessed the differentiation outcomes of NSCs transplanted with varying concentrations of hydrogels. ≈30% of the grafted NSCs differentiated into Tubulin beta 3 (Tubb3)‐positive neuronal lineage, while a similar percentage exhibited Glial fibrillary acidic protein (GFAP)‐positive astrocytic differentiation (**Figure** **3**A,B). There were no significant differences between the groups with 10% and 16% hydrogels. Our previous study demonstrated that the fibrotic microenvironment within the hydrogel‐created ECM hinders host axons from growing into the newly assembled matrix.^[^ ^45^ ^]^ We evaluated the expression of fibrotic proteins by quantifying fibronectin immunoreactivity and found no significant difference between the two groups (Figure S5A,B, Supporting Information). In addition, there were no notable differences in the extent of Picrosirius staining, which visualizes total collagen fibrils (Figure S5A,C, Supporting Information). These results suggest that neither the higher percentage of hydrogel nor the enhanced survival NSC grafts significantly affected the formation of fibrotic microenvironment. When we visualized the raphespinal axons from the brainstem serotonergic neurons, the growth of 5‐hydroxytryptamine (5‐HT) axons into the grafts was primarily confined to the regions near the boundary between the grafts and the host spinal cord (Figure 3C).

The quantification of axon lengths growing within the GFP‐positive grafts showed a trend toward an increase in animals that received NSCs in combination with 16% hydrogel. However, there was no statistically significant difference between the two groups (Figure 3D). We also examined the interaction between the 5‐HT axons growing into the grafts and GFP‐positive NSCs. The number of these interactions, reflected by the colocalization of 5‐HT and GFP immunoreactivities (arrows in Figure 3C), was significantly higher in the 16% hydrogel group than the 10% group (Figure 3E), probably due to a higher number of surviving NSCs in this group. The 5‐HT axons appeared to establish synaptic contacts with the GFP‐positive cells, as we observed a close apposition between the GFP+ cells and the 5‐HT axon terminals, which were colocalized with a presynaptic marker, synaptotagmin (Figure 3F–H). Collectively, these results indicate that enhance survival of NSCs via 16% hydrogel improved synaptic integration of transplanted NSCs.

### 2.4 Influence of the Substrate Stiffness on the Adhesive Properties and the Viability of Cultured NSCs

Hydrogels with varying concentrations may affect the survival of neural stem cells (NSCs) independently of changes in mechanical stiffness. For instance, different concentrations of hydrogels could influence degradation rates or the speed of gelation,^[^ ^46^ ^]^ both of which are crucial for encapsulating or protecting NSCs within the hydrogel. To examine whether different hydrogel concentrations affect degradation rates in vivo, we subcutaneously injected 10% and 16% I‐5 hydrogels mixed with a fluorescent dye into the left and right dorsal skin of mice and monitored the fluorescence signals from the hydrogel (Figure S6, Supporting Information). The fluorescence intensity began to decrease as early as 3 days post‐injection and continued to decline until 14 days, when signals were barely detectable. However, we did not find any significant differences in the degradation kinetics between the 10% and 16% hydrogels (Figure S6, Supporting Information). Cryo‐SEM measurements showed that the pore sizes of the 10%, 13%, and 16% hydrogels tended to decrease slightly (3.19 ± 0.47, 2.66 ± 0.57, and 2.20 ± 0.44 µm, respectively) (Figure S7A, Supporting Information). Previous studies reported that differences in micro‐scale pore size do not affect cell survival.^[^ ^47^ *^,^* ^48^ ^]^ The chemical composition of I‐5 hydrogel was characterized by FT‐IR analysis, which confirmed that changes in polymer concentration did not alter the chemical structure of the hydrogel network (Figure S7B, Supporting Information). Moreover, polymerization kinetics showed no significant differences (Figure S7C, Supporting Information). We also compared the speed of gelation by visually inspecting the gelation of 10% and 16% I‐5 hydrogels mixed with dye injected into a jar containing warm water at 37 °C. The injected hydrogel sank to the bottom of the jar and transformed into a gel‐like material within a few seconds (Movie S1, Supporting Information). No noticeable difference in gelation speed was observed between the hydrogels of different concentrations. These data suggest that the variation in stiffness is the primary factor that can explain the observed differences in NSC survival when complexed with various concentrations of the I‐5 hydrogel.

**Figure 4:**
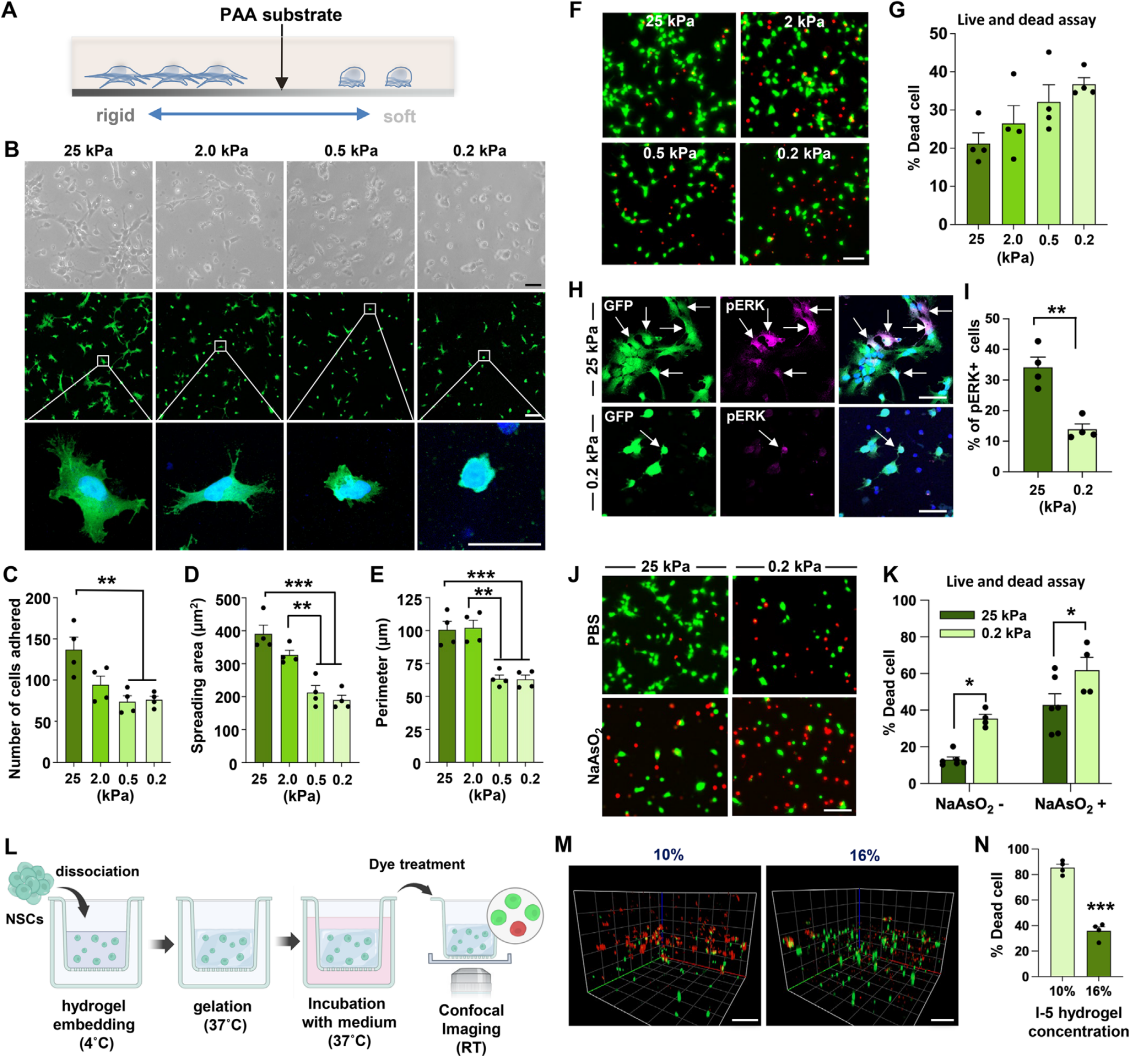
Influence of the substrate stiffness on the adhesive properties and the viability of cultured NSCs. A) A diagram depicting the in vitro culture system providing polymer matrix with varying degree of mechanical stiffness. PAA = polyacrylamide. B) Representative images of NSCs obtained during adhesion assay. NSCs expressing GFP were grown on substrates with mechanical stiffness ranging from 25 to 0.2 kPa. Upper panel: Phase‐contrast images. Middle panel: NSCs were visualized using GFP fluorescence. Boxed regions show magnified cell morphologies below. Scale bars = 20 µm. C–E) Quantitative graphs comparing the number of cells adhered (C), areas of cell spreading (D), and the perimeter of cell boundary (E). Each dot represents an independent culture replicate, with each replicate being an average value from three coverslips. Error bars represent SEM. ** and *** indicates *p* < 0.05 and *p* < 0.001 by one‐way ANOVA followed by Tukey's *post hoc* analysis. F,G) Representative images of NSCs for cell survival assay. NSCs were cultured on hydrogel substrates ranging from 25 to 0.2 kPa stiffness for 24 h. Subsequently, cell survival assay was performed. Live cells are labeled by Calcein‐AM (green), and dead cells are labeled by Ethidium Homodimer‐1 (red). Quantitative graph depicting the percentage of dead cells (G). Each dot represents an independent culture replicate, with each replicate being an average value from three coverslips. Error bars indicate SEM. Scale bars = 100 µm. H,I) Representative images of phospho‐ERK immunostaining (H). Arrows indicate GFP positive cells colocalized with phospho‐ERK (pERK, magenta) expression. The total number of NSCs was divided by pERK positive cells (I). Error bars represent SEM. ** indicates *p* < 0.01 by unpaired *t*‐test. Each dot represents an independent culture replicate, with each replicate being an average value from three coverslips. Scale bar = 50 µm. J,K) Representative images of NSCs for cell survival assay with sodium arsenite treatment. Sodium arsenite (NaAsO_2_) was used to induce cellular stress for 24 h (J). Quantitative graph of percentage of dead cells (K). N = 6 and 4 for 25 and 0.2 kPa groups, respectively. Error bars represent SEM. * indicates *p* < 0.05 by unpaired *t*‐test. Error bars represent the SEM. Scale bars = 100 µm. (L) Graphical illustration of the 3D hydrogel live/dead cell survival assay methods. (M) Representative confocal microscopic projection images of NSCs cultured in 3D hydrogels with different concentrations. Scale bar = 100 µm. (N) Quantification of dead cell percentages in 3D hydrogel cultures. *** indicates *p* < 0.001 by unpaired *t*‐test. N = 4 independent 3D cultures for both 10% and 16% hydrogel concentrations. Each dot represents an independent 3D culture replicate. Error bars represent SEM.

The results above led us to hypothesize that the mechanical properties of the hydrogel, determined by its concentration, could significantly influence the viability of NSCs surrounded by the hydrogel. To test this hypothesis in vitro, we utilized a culture model in which the mechanical stiffness of the substrate on which NSCs are grown is precisely controlled.^[^ ^49^ *^,^* ^50^ ^]^ NSCs were plated onto polyacrylamide (PAA) hydrogel coverslips with elastic moduli of 25, 2, 0.5, and 0.2 kPa (**Figure** **4**A,B).

We first examined the influence of hydrogel substrates with varying levels of stiffness on the adhesion and cellular morphology of cultured NSCs. There was a significant group difference in the number of NSCs attached onto substrates with different elastic moduli (*F*_(3, 12)_ = 8.198, *p* < 0.01), with the number of NSCs attached onto the most rigid substrate (25 kPa) significantly higher than that on softer substrates (0.5 and 0.2 kPa) (Figure 4B,C). Additionally, NSCs grown on softer substrates (0.5 and 0.2 kPa) exhibited a more round morphology with fewer cytoplasmic extensions, while NSCs grown on stiffer substrates (25 and 2 kPa) displayed a polygonal shape with more prominent processes. To quantitatively compare the differences in cellular morphology, we measured the spreading area and perimeter of adhered NSCs. Significant differences were observed between the groups for both measurements: *F*_(3, 12)_ = 22.63, *p* < 0.001 for spreading area, and *F_(_*_3, 12)_ = 20.88, *p* < 0.001 for perimeter. *Post hoc* analysis indicated that both the spreading area and perimeter of NSCs on stiffer substrates (25 and 2 kPa) were significantly larger than those on softer substrates (0.5 and 0.2 kPa) (Figure 4D,E).

Next, we examined how substrate stiffness affects cellular viability. Cell viability was measured using calcein AM for live cells and ethidium homodimer‐1 for dead cells. We observed a trend toward an increase in the percentage of dead cells as stiffness decreased, with the highest percentage of dead cells found in the condition with the lowest stiffness (0.2 kPa) (Figure 4F,G). However, the differences between groups were not statistically significant (*F_(_*_3, 12)_ = 3.45, *p* = 0.0514). We compared the expression of pERK, a marker of NSC viability post‐transplantation, between NSCs grown on stiff (25 kPa) and soft (0.2 kPa) hydrogel substrates (Figure 4H,I). The percentage of NSCs expressing pERK was significantly higher on the 25 kPa substrate than on the 0.2 kPa substrate, indicating more robust activation of pro‐survival signals in NSCs on stiffer substrates. To assess the NSC viability in an environment that mimics injured spinal cord, we treated NSCs with sodium arsenite (NaAsO_2_) to induce cellular stress.^[^ ^51^ ^]^ The effects of substrate stiffness on NSC viability were significantly observed in the NaAsO_2_‐treated conditions (Figure 4J,K), similar to those without treatment (*F*_(1, 16)_ = 17.77, *p* < 0.001 by two‐way ANOVA), suggesting that the mechanical environment may also play a role in supporting NSC viability under conditions of cellular stress.

To examine the influence of the mechanical stiffness in conditions that more closely mimic the difference between 10% and 16% I‐5 hydrogel in vivo, we established a 3D in vitro culture system using I‐5 hydrogel, leveraging the temperature‐dependent gelation of this hydrogel (Figure 4L). NSCs were seeded into the hydrogel at a solution state at 4 °C and embedded following the gelation at 37 °C before adding the culture medium. NSCs grown within this environment, composed of 10% hydrogel, exhibited a low survival rate (high percentage of dead cells) even without inducing cellular stress (Figure 4M,N). However, growing NSCs in the 3D culture with much stiffer 16% hydrogel consistently and significantly reduced cell death, supporting the idea that a stiffer hydrogel mechanical environment promotes cellular survival.

### 2.5 Actin Polymerization Determines the Substrate Stiffness‐Dependent Cellular Elasticity and Intracellular Calcium Dynamics in NSCs

**Figure 5:**
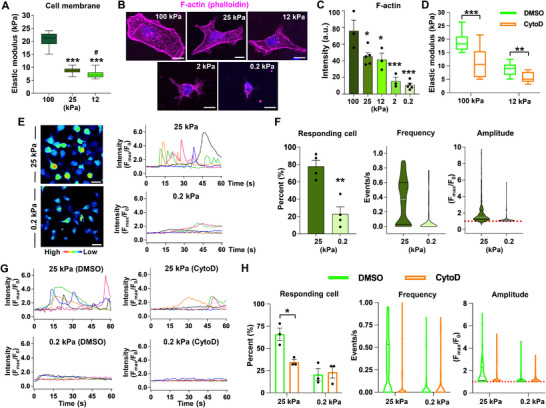
Actin polymerization determines the stiffness‐dependent cellular elasticity and intracellular calcium dynamics in NSCs. A) Nanoindentation analysis of the elastic modulus of NSCs cultured on hydrogel substrates with stiffness of 100, 25, and 12 kPa. Atomic force microscopy (AFM) was used to measure cellular membrane stiffness. Whiskers indicate the range of the minimum and maximum values. The lines within the boxes indicate the median values. *** indicates *p* < 0.001 compared to 100 kPa, and # indicates *p* < 0.05 compared to 25 kPa group by one‐way ANOVA followed by Tukey's *post hoc* analysis. N = 20, 25, and 20 cells for 100, 25, and 12 kPa, respectively. B,C) Representative images of filamentous actin (F‐actin) visualization in NSCs grown on 100, 25, 12, 2, and 0.2 kPa hydrogel substrates (B). F‐actin was stained using Alexa Fluor 594‐Phalloidin co‐labeled with DAPI. Quantification graph of actin staining intensity (C). Each dot represents a measurement of one or two coverslips from an independent culture. Error bars represent SEM. *** and * indicate *p* < 0.001 and *p* < 0.05, respectively, by one‐way ANOVA followed by Tukey's *post hoc* analysis. Error bars represent SEM. Scale bar = 5 µm. D) Changes in NSC plasma membrane elastic modulus measured by AFM following treatment with 20 µM of cytochalasine D (CytoD), actin destabilizer. N = 20 and 30 cells for 100 and 12 kPa (DMSO) groups, respectively, and N = 20 for both 100 and 12 kPa (CytoD) groups. Whiskers indicate the range of the minimum and maximum values. The lines within the boxes indicate the median values. *** indicates *p* < 0.001 and ** indicates *p* < 0.01 by two‐way ANOVA followed by Bonferroni's *post hoc* analysis. E) Intracellular calcium oscillations in NSCs grown on 25 kPa and 0.2 kPa hydrogel substrates. Left: representative calcium intensity maps (red: high level of Ca2+, blue: low level of Ca2+). Right: representative plots of calcium oscillations over a 60 s period. Each color represents an individual cell. F) Quantitative graphs of intracellular calcium oscillation parameters: responding cells (%), frequency (events s^−1^), and amplitude (F_max_/F_0_). ** indicates *p* < 0.01 by unpaired *t*‐test. N = 200 and 190 cells from 4 independent cultures for 0.2 and 25 kPa groups. Each dot represents an average value from an independent culture. G) Intracellular calcium oscillation after cytochalasin D (CytoD) treatment. H) Quantification graphs of intracellular calcium oscillation parameters: responding cells (%), frequency (events s^−1^), and amplitude (F_max_/F_0_). * indicates *p* < 0.05 by unpaired *t*‐test. N = 150 cells for 0.2 kPa + DMSO; N = 130 cells for 0.2 kPa + cytoD; N = 150 cells for 25 kPa + DMSO; and N = 150 cells for 25 kPa + cytoD group. Three independent cultures were conducted. Error bars represent SEM. Each dot represents an average value from an independent culture.

If the mechanical stiffness of a hydrogel substrate affects various cellular behaviors, it should also influence the mechanical properties of NSCs.^[^ ^52^ ^]^ We compared the cellular elasticity of NSCs grown on hydrogel substrates with varying mechanical stiffness using atomic force microscopy (AFM)^[^ ^53^ ^]^ (Figure S8A, Supporting Information). We first measured the elastic modulus (EM) of the PAA hydrogel substrates. The AFM used for this study could measure the EM of the 12 kPa substrate and higher, but not detect any measurable elasticity of the substrates softer than 12 kPa. The measured EM values for the substrates with stiffness levels of 100, 25, and 12 kPa, as designated by the manufacturer, closely aligned with those provided by the manufacturer (Figure S8B, Supporting Information). NSCs cultured on a substrate with 100 kPa exhibited ≈20 kPa elasticity (**Figure** **5**A), and the cellular elasticity is decreased proportionally on softer substrates. Since filamentous actin (F‐actin) is a major determinant of cellular elasticity,^[^ ^54^ ^]^ we visualized the F‐actin using the phalloidin conjugated with a fluorescent dye. The F‐actin signals were notably intense along the NSC membrane on the 100 kPa substrate (Figure 5B).

In these cells, F‐actin fibers appeared as linearly running bundles, reminiscent of stress fibers, within the cytoplasmic compartment. The F‐actin signals were slightly, but significantly decreased in NSCs grown on the 25 and 12 kPa substrates, and there was no obvious difference between cells on the two different substrates. In contrast, discernible F‐actin fibers were barely detectable in the submembranous or intracellular compartments in NSCs on either the 2 or 0.2 kPa substrate, resulting in a significant reduction of the F‐actin signal intensity (Figure 5B,C). There was no noticeable difference in the F‐actin signals between these two substrates with low EM values. To test if actin polymerization plays a role in the stiffness‐dependent elasticity, NSCs on either 100 or 12 kPa substrates were treated with the actin depolymerizing agent, cytochalasin D (cytoD). CytoD effectively disrupted the F‐actin fiber network in NSCs in a dose‐dependent manner (Figure S9, Supporting Information). The cellular elasticity of NSCs cultured on the 100 kPa substrate was reduced to nearly half following cytoD treatment (Figure 5D), and CytoD treatment in the 12 kPa condition also significantly decreased cellular elasticity. However, the extent of the drug's effect was less pronounced compared to NSCs grown on the stiffer substrate. A two‐way ANOVA analysis revealed that both the drug treatment and substrate stiffness had statistically significant effects on the EM of NSCs (drug treatment, *F*_(1, 86)_ = 52.1, *p* < 0.001; stiffness, *F*_(1, 86)_ = 111.5, *p* < 0.001). Furthermore, the interaction between the two factors was also statistically significant (*F*_(1, 86)_ = 10.0, *p* < 0.01), suggesting that actin depolymerization had a greater effect on cellular elasticity in NSCs grown on stiffer substrates.

The mechanical environment can modulate intracellular calcium ion (Ca^2+^) oscillations.^[^ ^55^ *^–^* ^57^ ^]^ In addition, the dynamics of the actin cytoskeleton are closely linked to these Ca^2+^ oscillations.^[^ ^58^ *^,^* ^59^ ^]^ Therefore, we examined whether the intracellular Ca^2+^ oscillations in NSCs are differentially regulated depending on the stiffness levels of a hydrogel substrate on which NSCs are grown. Many NSCs grown on a plastic dish exhibited spontaneous Ca^2+^ spikes, visualized by Fluo‐4 AM, lasting for several seconds, and these spikes frequently oscillated during the 1‐min imaging session (Movie S2, Supporting Information). These spontaneous Ca^2+^ oscillations were also observed to a similar extent in NSCs grown on the 25 kPa substrate (Figure 5E, Movie S3, Supporting Information), with almost 80% of NSCs showing at least one transient Ca^2+^ wave. In contrast, the Ca^2+^ activities were largely attenuated in NSCs on 0.2 kPa substrate with a significantly lower number of Ca^2+^ spikes observed in the 0.2 kPa condition (Figure 5F). The frequency and the amplitude of observed Ca^2+^ spikes were also markedly lowered in NSCs in the 0.2 kPa than those in the 25 kPa condition (25 kPa versus 0.2 kPa; frequency, 0.36 s^−1^ ± 0.29 versus 0.08 s^−1^ ± 0.19; amplitude, 0.98 ± 1.28 versus 0.25 ± 0.51). Next, we determined whether the mechanical stiffness‐dependent intracellular Ca^2+^ oscillations are mediated by actin polymerization. CytoD treatment markedly attenuated the oscillatory Ca^2+^ activities in NSCs grown on the 25 kPa substrate (Movies S4, Supporting Information), while NSCs on the 0.2 kPa substrate did not show considerable changes in response to cytoD treatment (Figure 5G). A two‐way ANOVA analysis revealed that both the drug treatment and substrate stiffness had statistically significant effects on the number of responding NSCs (drug treatment, *F*_(1, 8)_ = 5.654, *p* < 0.05; stiffness, *F*_(1, 8)_ = 22.09, *p* < 0.01). The interaction between the two factors was statistically significant (*F*_(1, 8)_ = 7.954, *p* < 0.05), indicating that cytoD treatment significantly influenced Ca^2+^ oscillation activity in NSCs grown on stiffer substrates (Figure 5H). In the 25 kPa condition, the frequency and amplitude of Ca^2+^ spikes were substantially reduced by cytoD in NSCs (DMSO vehicle versus cytoD; frequency, 0.30 s^−1^ ± 0.31 versus 0.11 s^−1^ ± 0.23; amplitude, 1.84 ± 1.01 versus 1.48 ± 0.80). In contrast, cytoD treatment did not produce significant changes in the 0.2 kPa condition.

### 2.6 Involvement of Piezo1 in the Stiffness‐Dependent Adhesive Behavior of Cultured NSCs

**Figure 6:**
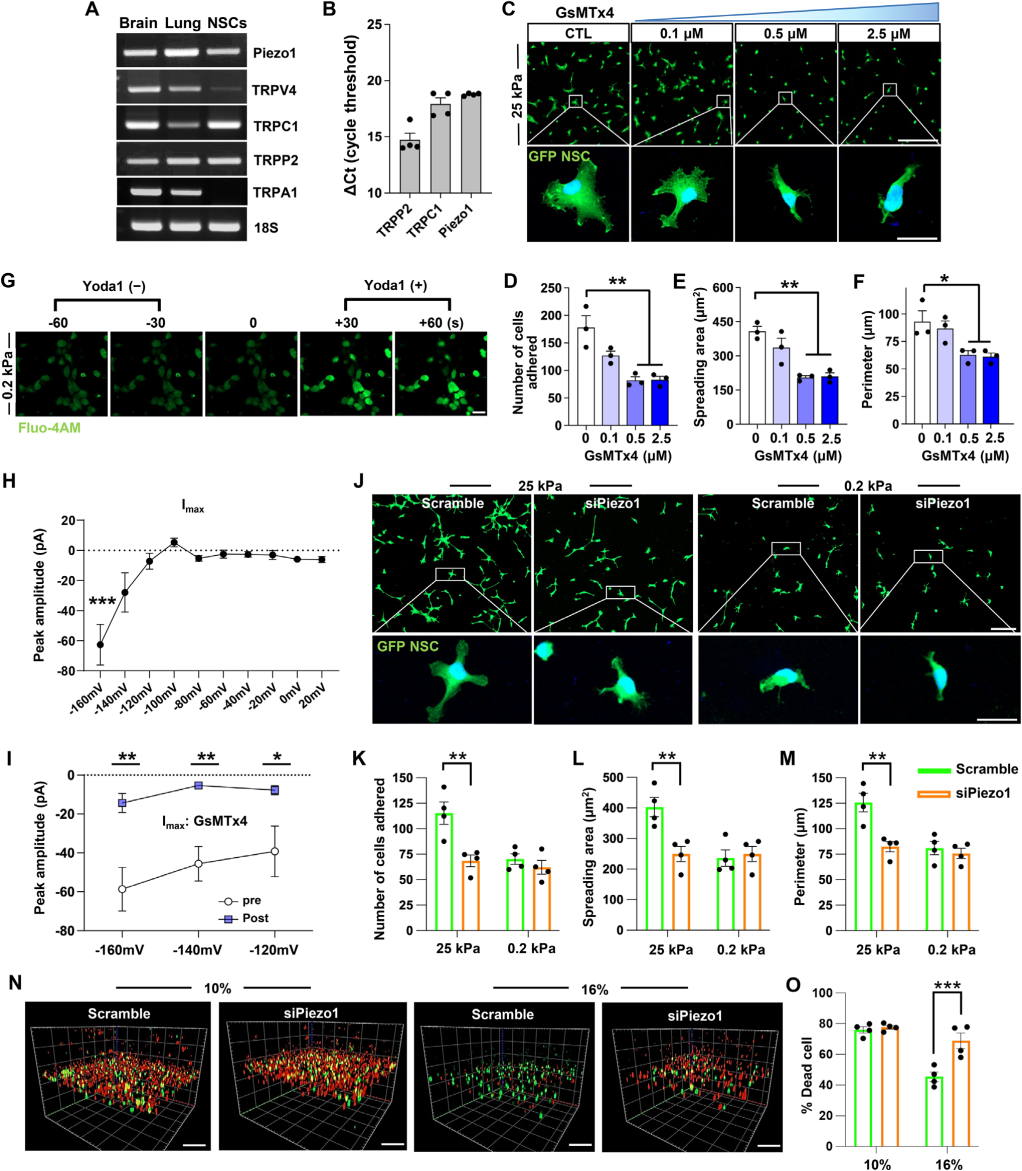
Involvement of Piezo1 in the stiffness‐dependent adhesive behavior of cultured NSCs A) Representative mRNA expression of putative mechanosensitive ion channels in NSCs. Brain and lung tissues were used as positive controls for RT‐PCR products. 18S rRNA was used as an internal reference. B) Quantitative measurement of mRNA expression by qRT‐PCR. Each dot represents an independent biological replicate. Error bars represent SEM. C–F) NSC adhesion assay with GsMTx4. NSCs were treated with GsMTx4 for 24 h on 25 kPa hydrogel substrate (C). Quantitative graphs comparing the number of cells adhered (D), areas of cell spreading (E), and the perimeter of cell boundary (F). Error bars represent SEM. ** indicates *p* < 0.01 and * indicates *p* < 0.05 by one‐way ANOVA followed by Tukey's *post hoc* analysis. Three biological replicates were performed. Boxed regions are magnified below. Scale bar = 20 µm. G) Representative images of Piezo1 functional assay in NSCs treated with the Piezo1 agonist (Yoda1). NSCs were grown on 0.2 kPa hydrogel substrates, and intracellular calcium uptake was visualized using Fluo4‐AM. Piezo1 agonist (Yoda1) was added 60 s after baseline recording. Scale bar = 20 µm. H) Quantification of the peak amplitude of the inward currents (I_max_) at varying holding potentials. *** indicate *p* < 0.001 compared to the I_max_ at 0 mV holding potential by one way ANOVA followed by Tukey's *post hoc* analysis. Each data point represents the average amplitude from 10 independent measurements using 10 cells (N = 10). Error bars represent SEM. I) Comparison of I_max_ before and after treatment with a Piezo1 blocker, GsMTx4, at a concentration of 5 µM. I_max_ values were obtained before drug treatment and 20 min after drug treatment from 7 independent cells (N = 7). Error bars represent SEM. * and ** indicate *p* < 0.05 and *p* < 0.01 by two‐way ANOVA followed by *post hoc* Bonferroni test. J–M) NSC adhesion assay with Piezo1 knockdown by electroporation. Scrambled siRNA was used as a control (J). Quantitative graphs comparing the number of cells adhered (K), areas of cell spreading (L), and the perimeter of cell boundary (M). Boxed regions are magnified below. ** indicates *p* < 0.01 by two‐way ANOVA followed by *post hoc* Bonferroni test. Each dot represents an independent culture replicate, with each replicate being a measurement from one coverslip. Error bars represent SEM. Scale bar = 20 µm. N) Representative confocal microscopic projection images of NSC live/dead cell survival in 3D hydrogels with different concentrations following Piezo1 knockdown. Scale bar = 100 µm. O) Quantitative graph comparing dead cell percentage in 3D hydrogel culture (N = 4 independent 3D cultures per group). *** indicates *p* < 0.001 by two‐way ANOVA followed by *post hoc* Bonferroni test. Each dot represents an independent 3D culture replicate. Error bars represent SEM.

Mechanosensitive ion channels translate changes in a mechanical environment into ionic flow, responding to extracellular physical cues and influencing various fundamental physiological processes at both the organismal and cellular levels.^[^ ^60^ ^]^ We screened the expression of *Piezo1* and various transient receptor potential (TRP) channels in NSCs, which are mechanosensitive ion channels known to be expressed in mammalian stem cells.^[^ ^61^ ^]^ Substantial mRNA expression of *Piezo1*, *TRPC1*, and *TRPP2*, but not *TRPV4* and *TRPA1*, in NSCs were observed compared to the rat brain and lung tissues as positive controls (**Figure** **6**A). Among these, *TRPP2* had the highest expression level in NSCs, followed by *TRPC1* and *Piezo1* (Figure 6B). To examine the functional implications of these mechanosensitive ion channels in the adhesive properties of cultured NSCs (see Figure 4B–E), NSCs grown on a stiff 25 kPa substrate were treated with various inhibitors targeting these mechanosensitive channels. First, amiloride hydrochloride, a TRPP2 channel blocker, did not significantly impact the number of adhered NSCs at a concentration of up to 1 µM, but 10 µM of amiloride hydrochloride slightly decreased the number of adhered cells. A significant group difference was observed (*F*_(3, 8)_ = 5.850, *p* < 0.05 by one‐way ANOVA), and *post hoc* analysis showed a significant decrease by amiloride hydrochloride only at 10 µM concentration (Figure S10A–D, Supporting Information). However, the spreading area and perimeter, which are parameters of cultured NSC morphology, were not affected (Figure S10C,D, Supporting Information). Treatment with Pico145, a specific inhibitor of TRPC1/4/5, did not influence the number or morphology of adhered NSCs, even at the highest concentration (Figure S10E–H, Supporting Information), ruling out the potential involvement of TRPC1. In contrast, when GsMTx4, an inhibitor of mechanosensitive ion channels belonging to the Piezo and TRP families was treated, the polygonal shape of NSCs, characterized by notable cellular branches, became rounder with fewer processes in response to higher concentrations of GsMTx4 (Figure 6C), similar to NSCs on a softer substrate (see Figure 4B).

A significant group difference in the number of adhered NSCs was observed between different concentrations of GsMTx4 treatment (*F*_(3, 8)_ = 13.60, *p* < 0.01), and *post hoc* analysis showed significant decreases by GsMTx4 at 0.5 and 2.5 µM concentrations (Figure 6D). There were significant differences also in both the spreading area and perimeter among the groups: *F*_(3, 8)_ = 16.26, *p* < 0.001 for spreading area, and *F_(_*_3, 8)_ = 6.189, *p* < 0.05 for perimeter. *Post hoc* analysis revealed a similar result to that from the cell number analysis (Figure 6E,F). Thus, we concluded that Piezo1 was the primary mechanosensitive ion channel responsible for the adhesive properties of NSCs on a stiff substrate among those that were substantially expressed in NSCs. To verify the functionality of the Piezo1 channel in NSCs, we performed a calcium imaging experiment after treating NSCs grown on a 0.2 kPa substrate with the Piezo1 agonist Yoda1. The treatment with Yoda1 resulted in significant and sustained Ca^2+^ influxes (Figure 6G and Movie S5, Supporting Information), demonstrating that the Piezo1 channel is indeed functional in NSCs.

To provide direct evidence that mechanical stimulation of NSCs induces ionic currents through mechanosensitive channels, we conducted whole‐cell patch‐clamp recordings on cultured NSCs. We activated the mechanosensitive ion channels by applying short air pulses at 7 psi positive pressure, lasting 10 ms, using a Picospritzer device and measured inward current using a voltage‐clamp mode (Figure S11, Supporting Information). Notably, inward deflection currents in response to the pressure stimulation began to appear at −120 mV holding potentials and became more robust at higher holding potentials. The mean peak amplitude of the inward currents, I_max_, at −160 mV holding potential was ≈60 pA and significantly larger than that at 0 holding potential (Figure 6H). To further confirm that the observed inward currents were mediated by the Piezo1 channel, we treated NSCs with a Piezo1 blocker, GsMTx4, and compared the I_max_ values before and after the treatment. The treatment with GsMTx4 significantly reduced the magnitude of I_max_ at −120, −140, and −160 mV holding potentials (Figure 6I), demonstrating that the pressure‐induced currents were dependent on the activity of the Piezo1 channel.

To examine the role of Piezo1 in the stiffness‐dependent adhesive properties of cultured NSCs, Piezo1 expression was suppressed using siRNA. The levels of Piezo1 expression in NSCs were comparable regardless of whether they were cultured on a standard plastic substrate or on PAA substrates with stiffness values of 25 and 0.2 kPa (Figure S12A, Supporting Information). When siRNA targeting Piezo1 was treated to NSCs grown on a 25 kPa substrate, the NSCs underwent a significant morphological change from a polygonal shape to a more rounded appearance, which included a loss of cytoplasmic processes (Figure 6J). The morphological changes were accompanied by a decrease in the number of adhered cells in the siPiezo group. In contrast, the morphology and number of NSCs cultured on a 0.2 kPa substrate showed no significant changes following Piezo1 knockdown. A two‐way ANOVA analysis revealed that both the siPiezo1 treatment and substrate stiffness had statistically significant effects on the number of adhered NSCs (siPiezo1, *F*_(1, 12)_ = 13.32, *p* < 0.01; stiffness, *F*_(1, 12)_ = 11.71, *p* < 0.01) (Figure 6K). Furthermore, the interaction between the two factors was also statistically significant (*F*_(1, 12)_ = 6.604, *p* < 0.05), indicating that Piezo1 knockdown had a more significant effect on NSCs grown on a stiffer substrate. Statistical analyses of the spreading area and perimeter showed similar results (Figure 6L,M), revealing a significant influence of siPiezo1 on the morphology of NSCs on a stiffer substrate. We further assess the influence of Piezo1 on the survival of NSCs grown in the 3D hydrogel cultures illustrated in Figure 4L. The majority of NSCs in the 3D culture composed of 10% I‐5 hydrogel showed evidence of cell death, and the percentage of dead cells was not significantly affected by the knockdown of Piezo1 by siRNA (Figure 6N,O). As shown in Figure 4M, the survival of NSCs in 16% 3D culture was substantially improved, and the enhanced survival was significantly compromised when siRNA against Piezo1 was treated. A two‐way ANOVA revealed that both the siPiezo1 treatment and hydrogel stiffness had statistically significant effects on the percent dead cells (siPiezo1, *F*_(1, 12)_ = 15.61, *p* < 0.01; hydrogel concentration, *F*_(1, 12)_ = 37.78, *p* < 0.001). Furthermore, the interaction between the two factors was also statistically significant (*F*_(1, 12)_ = 11.56, *p* < 0.01), indicating that Piezo1 plays an essential role in hydrogel stiffness‐dependent NSC survival in 3D cultures composed of I‐5 hydrogel.

### 2.7 CRISPR/Cas9‐Mediated *Piezo1* Gene Editing Abolishes the Stiffness‐Dependent Survival of NSC Grafts in the Injured Spinal Cord

**Figure 7:**
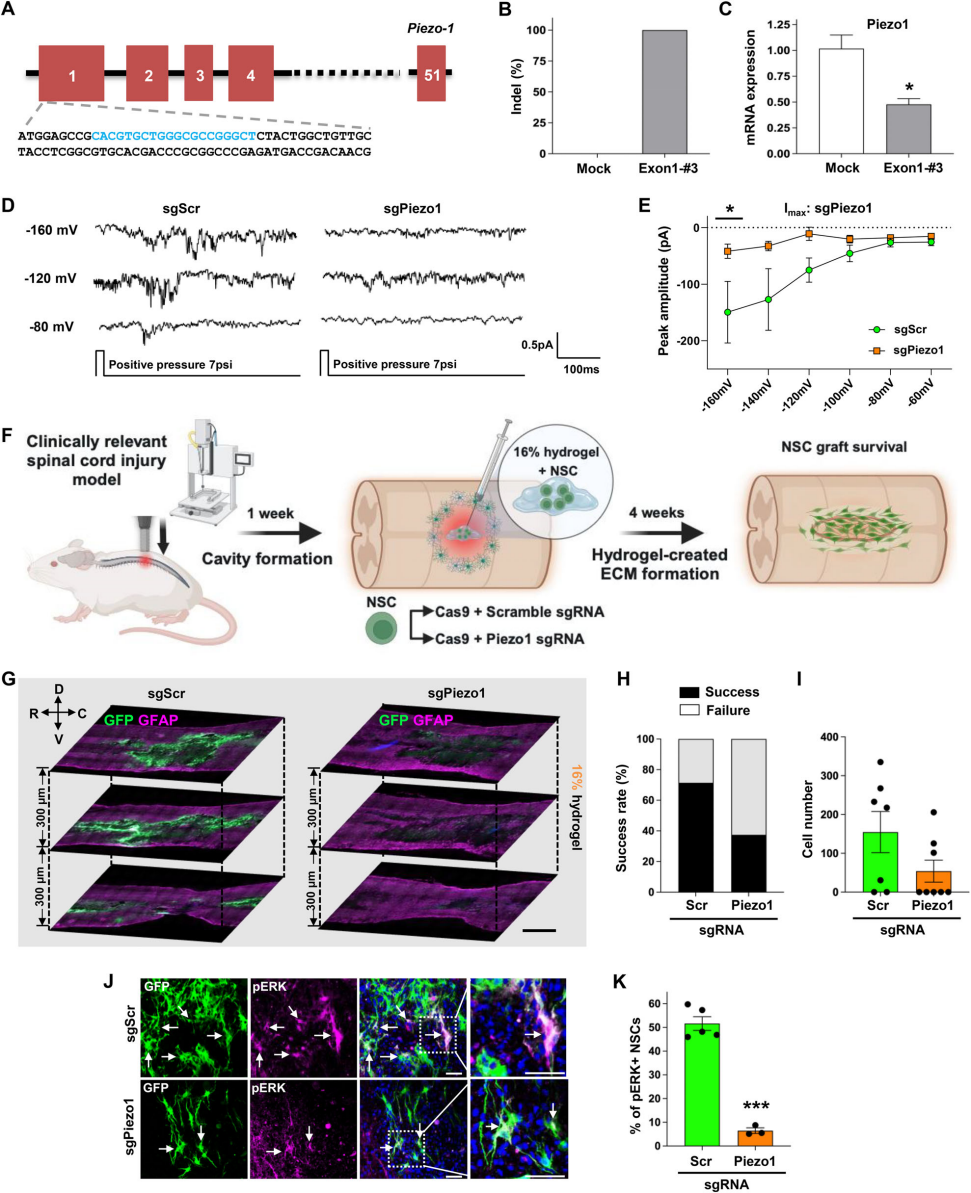
CRISPR/Cas9‐mediated *Piezo1* gene editing abolishes the stiffness‐dependent survival of NSCs in the injured spinal cord. A) DNA sequences (shown in blue) in the proximal exon1 of the *Piezo1* gene targeted by the chosen sgRNA. B) Indel frequency of Crispr/Cas9 gene editing using the chosen sgRNA sequence (exon1‐#3). C) Measurement of *Piezo1* mRNA levels by quantitative RT‐PCR. * indicates *p* < 0.05 by unpaired *t*‐test (N = 3 replicates per group). Error bars represent SEM. D) Representative raw current traces from NSCs with control scrambled sgRNA (sgScr) and sgPiezo1 at varying holding potentials. E) Quantification of the peak amplitude of the inward currents (Imax) at varying holding potentials. * indicates *p* < 0.05 by two‐way ANOVA followed by Bonferroni's *post hoc* analysis. N = 5 cells per group. Error bars represent SEM. F) Schematic diagram illustrating the experimental design for Crispr/Cas9‐mediated long‐term Piezo1 knockdown in vivo study. G) Representative images of longitudinal spinal cord sections obtained from animals transplanted with NSCs edited by Crispr/Cas9 (sgPiezo1) or control (sgScr). Three longitudinal spinal cord sections containing the lesion epicenter are displayed with a 300 µm interval along the dorsal‐ventral (D‐V) axis. R indicates rostral and C caudal direction. GFP indicates surviving NSCs (green) and GFAP (magenta) demarcates lesion areas. Scale bar = 1000 µm. H,I) Quantitative graphs comparing the rate of graft success (H) and the number of GFP‐positive NSCs (I). Graft success was defined as the presence of noticeable GFP‐positive NSC grafts in at least one tissue section. N = 7 and 8 animals for sgScr and sgPiezo1 group, respectively. Error bars represent SEM. J) Representative images of phospho‐ERK immunostaining with surviving NSC grafts. Boxed regions are magnified on the right side. Scale bar = 50 µm. K) Quantitative graph showing the proportion of GFP and phospho‐ERK double‐positive cells. *** indicates *p* < 0.001 by unpaired *t*‐test. Error bars represent SEM. N = 5 and 3 animals for sgScr and sgPiezo1.

The above findings led us to hypothesize that the improved survival of NSCs transplanted in a complex with a higher percentage of I‐5 hydrogel may be attributed to the activation of Piezo1 channels in a stiffer mechanical environment. Since we found that siRNA‐mediated knockdown of Piezo1 did not last longer than a couple of days (Figure S12B, Supporting Information), we employed CRISPR/Cas9 gene editing for the long‐term suppression of *Piezo1* in primary rat NSCs. To target rat *Piezo1*, we searched for protospacer adjacent motif (PAM) sequences (NGG for *Streptococcus pyogenes* Cas9) within the protein coding sequences of rat *Piezo1* and designed 13 different single guide RNAs (sgRNAs) to target this region (Table S1, Supporting Information).The activities of these sgRNAs were screened after transfection of sgRNA‐Cas9 RNP complexes into a rat C6 glioma cell line and selected six sequences that demonstrated an indel frequency greater than 90% using targeted deep sequencing (Figure S13A, Supporting Information). We then performed RT‐qPCR and selected the sgRNA sequence #3 in exon1 of the *Piezo1* (lead sgRNA labeled as sgPiezo1 hereafter) based on the knockdown efficiency (Figure S13B, Supporting Information and **Figure** **7**A).

Using this sgRNA, we achieved near 100% indel frequency (with most frequent edits are >38 bp deletions; Table S2, Supporting Information) and more than 50% reduction of *Piezo1* mRNA in primary NSCs (Figure 7C).

The functional suppression of the Piezo1 channel was assessed by visualizing Ca^2+^ influx induced by Yoda1. Treatment with Yoda1 resulted in a significant increase in Ca^2+^ influx in NSCs grown on 0.2 kPa substrate that were transfected with either Cas9 alone or Cas9 in conjunction with a control scrambled sgRNA (sgScr) (Figure S12A, Supporting Information). However, the Ca^2+^ influx induced by Yoda1 was noticeably reduced in NSCs that were transfected with both Cas9 and sgPiezo1 (Figure S14A–C, Supporting Information). To investigate whether CRISPR/Cas9‐mediated editing of the *Piezo1* gene effectively inhibited ionic currents triggered by mechanical stimulation, we measured inward ionic currents in response to pressure stimulation in *Piezo1*‐edited NSCs. In NSCs with control sgScr, we observed distinctive inward currents at holding potentials of −120 and −160 mV (Figure 7D), but these currents almost disappeared in NSCs with sgPiezo1. Quantitative analysis showed a significant reduction in the I_max_ at the −160 mV holding potential in NSCs with sgPiezo1 (Figure 7E), indicating the *Piezo1* gene editing successfully suppressed functional mechanosensitive channels.

To determine whether Piezo1 plays a crucial role in enhancing graft survival when NSCs are injected in combination with 16% I‐5 hydrogel (see Figure 2B,E), we conducted an in vivo experiment in which NSCs transfected with sgScr or sgPiezo1 were transplanted as a complex with the 16% I‐5 hydrogel 1 week after an initial injury (Figure 7F). A total of 15 animals were generated with 7 animals for sgScr and 8 animals for sgPiezo1, respectively, and these animals underwent survival analysis 4 weeks after transplantation. NSC grafts were observed in 5 out of 7 animals (71.4%) with control sgScr, and the majority of animals with graft success showed a sizable engraftment (Figure 7G–I). However, the rate of graft success was only 37.5% (3 out of 8 animals) in sgPiezo1 group, and the average number of surviving NSCs tended to be smaller in this group (Figure 7G–I). We counted the number of pERK positive NSCs in animals with graft success and discovered that the proportion of pERK positive NSCs was significantly reduced in animals with *Piezo1* gene editing (Figure 7J,K).

## 3 Discussion

This study was motivated by the observation that transplanting NSCs complexed with 10% I‐5 hydrogel did not enhance their survival, even though the hydrogel consistently prevented cavity formation and facilitated the creation of ECMs at the lesion epicenter. Our findings indicate that NSC graft survival significantly improved when they were transplanted with a stiffer (16%) I‐5 hydrogel compared to a softer (10%) hydrogel. By using an in vitro model with precisely controlled mechanical stiffness of the hydrogel substrate, we demonstrated that the mechanical properties of the hydrogel substrate substantially influence NSC behavior and viability accompanied by regulation of cellular elasticity and intracellular Ca^2+^ oscillations in NSCs via an actin polymerization‐dependent manner. We further showed that the mechanosensitive ion channel Piezo1 as the key mediator of these stiffness‐dependent behaviors. Importantly, stiffness‐dependent NSC survival in the injured spinal cord was attenuated by Crispr/Cas9‐based editing of the *Piezo1* gene in transplanted NSCs, highlighting the critical role of mechanotransduction in NSCs in modulating the viability of grafted NSCs in the injured spinal cord.

Despite the potential of NSC transplantation for repairing damaged spinal cord, poor graft survival remains a significant challenge.^[^ ^5^, ^6^, ^7^, ^8^, ^9^ ^]^ Recent studies have shown that using a combination of multiple growth factors and anti‐apoptotic molecules at high concentrations could dramatically improve graft survival.^[^ ^3^ ^]^ However, it remains to be determined whether this approach could result in undesirable off‐target effects, such as allodynia and autonomic dysreflexia, due to nonspecific and unregulated stimulation of host circuits.^[^ ^1^, ^62^ ^]^ Advances in biomaterial engineering have offered potential solutions to enhance post‐transplantation cell survival. In this study, while our hydrogel engineered to conjugate the imidazole moiety successfully created eosin‐positive matrices at the lesion core, fulfilling its role as a scaffold, NSC survival remained poor when complexed with a 10% hydrogel. Interestingly, when we increased the hydrogel concentration to 16%, which made it nearly five times stiffer, we observed a dramatic improvement in NSC graft survival. In our replication experiment, we confirmed a positive correlation between the stiffness of the I‐5 hydrogel stiffness and both the success rate and the number of surviving NSC grafts. This finding was further supported by an increase in pERK expression in NSCs transplanted with the stiffer hydrogel, indicating enhanced prosurvival intracellular signaling. Importantly, the improved survival was not attributable to differences in hydrogel degradation kinetics, gelation speed, pore size, hydrogel chemistry, or effects on neuroinflammation, as these parameters remained consistent across different hydrogel concentrations, indicating that the mechanical properties of the hydrogel directly influence NSC survival. Our previous study demonstrated that the hydrogel‐created ECM begins to accumulate as early as 1 week after injection and continues to mature over the course of 4 weeks post‐injection.^[^ ^20^ ^]^ In addition, it has been reported that CNS tissue, including the spinal cord, becomes softer soon after injury.^[^ ^38^ *^,^* ^63^ ^]^ Therefore, the lesioned spinal cord may be significantly softer in the days immediately following hydrogel injection than the uninjured spinal cord. During these early days of injection, the viability of NSCs may be highly influenced by the mechanical environment afforded by I‐5 hydrogel, which serves as a scaffolding platform before it begins to degrade completely. While substrate stiffness is known to shape NSC differentiation from previous in vitro studies,^[^ ^64^ *^–^* ^66^ ^]^ our study found no evident relationship between hydrogel stiffness and the fate of transplanted NSCs in the injured spinal cord. Unlike simplified in vitro systems, the injured spinal cord is highly dynamic, shaped by inflammation, reactive gliosis, and diverse paracrine signals that can override or mask stiffness‐mediated cues. This complexity makes it challenging to detect clear stiffness‐dependent effects on NSC lineage specification. Thus, while substrate stiffness strongly influences NSC differentiation under controlled in vitro conditions, this influence may be masked in the complex milieu of the injured spinal cord.

Our in vitro studies using PAA hydrogel substrates with defined elastic moduli provided mechanistic insights into how substrate stiffness affects NSC behavior. NSCs cultured on stiffer substrates (25 kPa) showed improved adhesion, displayed more complex morphology with extended processes, and exhibited higher viability compared to those on softer substrates (≈0.2–0.5 kPa). Cell‐matrix interaction in engineered polymer scaffolds exerts influence on cellular morphology.^[^ ^67^ *^,^* ^68^ ^]^ Increasing the stiffness of flat hydrogel surfaces led to enhanced spreading of mesenchymal stem cells,^[^ ^69^ ^]^ resulting in larger spreading areas, similar to the responses observed with NSCs grown on stiffer substrates in our study. Cellular contact with a stiffer polymer substrate may enhance the activity of the focal adhesion kinase,^[^ ^21^ ^]^ which in turn regulates the extent of cellular adhesion to the substrate. In addition, mechanotransduction involves various adaptive cellular responses, including mechanisms of proliferation and cell death.^[^ ^21^ ^]^ Our experiment demonstrated that stiffness‐dependent viability persists under cellular stress, indicating that a stiffer hydrogel could effectively protect NSCs in the degeneration‐prone environment of the injured spinal cord. We measured elasticity and intracellular Ca^2+^ oscillations as markers of active mechanotransduction on stiffer substrates. Both markers were highly dependent on actin polymerization, implying that the mechanotransduction primarily affects the cytoskeletal machinery beneath the cellular membrane. Intracellular Ca^2+^ oscillations within cells are crucial for various cellular processes, including survival and death.^[^ ^70^ ^]^ Consequently, stiffness‐dependent Ca^2+^ oscillatory activity may suggest a significant role of mechanotransduction in maintaining cellular homeostasis.

Among various mechanosensitive ion channels expressed in NSCs, Piezo1 emerged as the primary mediator of stiffness‐dependent cellular responses in the current study. Inhibition of Piezo1 with GsMTx4 or siRNA knockdown significantly altered NSC morphology and reduced adhesion, specifically on stiffer substrates, mimicking the behavior of NSCs on soft substrates. Electrophysiological recordings confirmed functional Piezo1 channels in NSCs, with pressure‐induced inward currents that were blocked by GsMTx4. Importantly, CRISPR/Cas9‐mediated *Piezo1* gene editing significantly reduced NSC graft survival in vivo when transplanted with 16% hydrogel, with success rates dropping from 71.4% to 37.5%. These findings establish Piezo1 as a critical mechanotransducer that senses the stiffness of the surrounding hydrogel environment and regulates NSC survival. Previous studies highlighted the role of Piezo1 in various stem cell functions, including fate differentiation and fate determination.^[^ ^37^ *^,^* ^61^ ^]^ Activation of Piezo1 in certain tumors enables them to evade apoptosis, thereby contributing to the survival and progression of cancerous cells.^[^ ^71^ *^,^* ^72^ ^]^ Our study appears to be the first report on the role of Piezo1 in regulating the survival of NSCs. Based on a variety of research focused on Piezo1 functions, we can infer that the activation of Piezo1 in transplanted NSCs, caused by the surrounding stiffer hydrogel, can initiate intracellular prosurvival signals, which in turn prevent the loss of NSCs during the early post‐transplantation period.

Our findings may have profound implications for cell‐based therapies across all central nervous system (CNS) injuries, not just spinal cord trauma. Optimizing the mechanical stiffness of hydrogels that encapsulate NSCs or other therapeutic cells could dramatically enhance graft survival, addressing one of the critical challenges in the field of cell‐based therapy. Our study also advances our understanding of cellular mechanotransduction in neural repair employing cell transplantation. Regardless of whether hydrogel is used, a suboptimal mechanical environment in the injured CNS may critically undermine the survival of grafted therapeutic cells. Injured spinal cord and brain tissues become significantly softer,^[^ ^63^ *^,^* ^73^ ^]^ likely due to ECM degradation, increased water content, and loss of myelination, among other factors. This stiffness mismatch between injured and intact tissue likely deprives transplanted cells of essential mechanotransduction‐dependent prosurvival signals. Since the physicochemical properties of hydrogels limit the extent to which their concentration can be increased, pharmacological activation of Piezo1 or genetic engineering to overexpress this channel could be an alternative approach to boost cell survival in mechanically suboptimal environments. However, it remains to be determined how the mechanical cues within hydrogels and the mechanotransduction mechanisms in NSCs instruct grafted cells to become functional neural cells that integrate with host neural circuits and contribute to functional recovery. Future research should explore the complex interplay between mechanical and biochemical cues in the injury microenvironment to develop comprehensive strategies that enhance both graft survival and functional integration.

## 4 Experimental Section

### Materials and Methods—Primary Neural Stem/Progenitor Cells Culture

NSCs were isolated from timed‐pregnant transgenic SD rats ubiquitously expressing enhanced green fluorescent protein (GFP) as previously described.^[^ ^6^ ^]^ Briefly, the spinal cords from E14 (embryo) fetuses of pregnant rats were dissected in a cold Hanks's Balanced Saline Solution (HBSS, Thermo) containing 1% antibiotics (Thermo) and antifungal (Thermo), and the meninges were carefully removed under the dissection microscope. Subsequently, the spinal cords were mechanically dissociated in a mixture of Accumax (Merck, #SCR006) and DNase I (Sigma, #10 104 159 001, 20 µg mL^−1^) and the dissociated cells were passed through a 40 µm cell strainer to remove debris followed by centrifugation for 5 min at 1000 RPM. The cells were resuspended in StemPro NSC SFM culture media (Thermo, #A1050901) containing basic fibroblast growth factor (bFGF, 20 ng mL^−1^) and epidermal growth factor (EGF,20 ng mL^−1^), and plated into a non‐coated Petri dish (SPL, #10 093) to grow as neurospheres in a humidified atmosphere with 5% CO_2_. The culture medium was changed every other day.

### Preparation of NSC‐Hydrogel Complex

In our previous studies,^[^ ^20^ *^,^* ^74^ ^]^ we have provided a comprehensive outline of the synthesis procedure for the I‐5 hydrogel. Briefly, poly(dichlorophosphazene) (10 g, 86.29 mmol) was sequentially reacted with isoleucine ethyl ester (23.81 g, 121.67 mmol), 2‐aminoethanol (1.56 g, 25.5 mmol), and aminopolyethylene glycol (56.29 g, 75.06 mmol) to poly(organophosphazene). Subsequently, the hydroxyl group of 2‐aminoethanol was esterified using succinic anhydride (2.76 g, 27.58 mmol) to create a hydrolysable ester linkage and a terminal carboxylic acid group. Finally, I‐5 was synthesized by conjugating 1–3 aminopropylimidazole (1.45 g, 11.54 mmol) to the carboxylic acid group. To prepare the NSC‐hydrogel complex, neurospheres were collected into 15 mL conical tubes by centrifugation for 1 min at 1000 RPM on the day of transplantation. Cell pellets were incubated in Accumax for 3 min at RT, followed by gentle mechanical dissociation into single cells. After centrifugation, cell pellets were resuspended at 5 × 10^5^ cells µL^−1^ in a culture medium and kept on ice until complexation with I‐5 hydrogel. Dissociated NSCs in a culture medium were mixed with an appropriate volume of I‐5 hydrogel in a solution state (20% stock polymer solution) to achieve final polymer concentrations of 10%, 13%, and 16%. To determine whether adding an exogenous growth factor or an extracellular matrix protein could improve graft survival, we included insulin‐like growth factor‐1 (R&D systems, #4326‐RG, 2 µg mL^−1^) or laminin (Thermo, #23 017 015, 3 µg mL^−1^) to the culture medium prior to the complexation with I‐5 hydrogel.

### Animals and Surgical Procedures

All animal protocols were approved (2020‐0005) by the Institutional Animal Care and Use Committee (IACUC) of Ajou University School of Medicine, South Korea. Adult female Sprague–Dawley (SD) rats (8 weeks) weighing 200–230 g (Orient Bio, South Korea) were used for all animal experiments. The SCI model was created by using the Infinite Horizon Impactor (Precision Systems and Instrumentation), as described in our previous studies.^[^ ^20^ *^,^* ^45^ ^]^ Briefly, the animals were deeply anesthetized by intraperitoneal injection of ketamine (80 mg kg^−1^) and xylazine (10 mg kg^−1^) mixtures. The hindlimb pedal withdrawal reflexes were examined to determine adequate anesthesia level before starting surgery. A longitudinal dorsal incision was created between T7‐T11 spinal vertebrae, followed by a laminectomy at the level of T9. Then, the subjects underwent a contusive SCI with a force of 200 kdynes, which replicates the clinically relevant pathophysiological events following SCI. Following surgery, animals were placed in a warming incubator at 37 °C until they were fully awake. Postoperative care included expressing the bladder twice daily until natural urination resumed. NSC transplantation was performed 1 week after SCI. The total number of transplanted NSCs per animal was 1 × 10^6^ cells in 10 µL of NSC‐hydrogel complex prepared as described above. The NSC‐hydrogel complex was loaded into a 26G Hamilton syringe (Hamilton #80 030) and kept on ice for at least 1 h until transplantation. It was crucial to maintain a temperature of 4 °C to ensure the solution state of the complex before transplantation. After re‐exposing the lesioned spinal cord, 10 µL of the NSC‐hydrogel complex was removed from the ice and injected into the lesion epicenter over 1 min without any delay. The syringe was then kept in place for 1 min to prevent leakage. Postoperative care was provided in the same way after the transplantation procedure.

### Assessment of the Physical Properties of I‐5 Hydrogel

Young's modulus was determined by conducting a temperature sweep from 5 to 60 °C at an oscillation frequency of 1 Hz and a strain of 10%. Frequency sweep tests were carried out across an angular frequency range of 0.1–100 rad s^−1^. Strain sweep was performed at a constant frequency of 1 Hz, over a strain range of 0.01% to 100%, to determine the linear viscoelastic region (LVR). Time sweep measurements were conducted at 1% strain and frequency 1 Hz for 600s to evaluate the temporal stability of the hydrogel structure. All frequency, strain, and time sweep analyses were conducted at a constant temperature of 37 °C using a rheometer (MSC 102, Anton Paar). Storage and loss moduli were obtained using the instrument software. Young's modulus (E) was estimated using the measured storage modulus (G′) and loss modulus (G″) from rheology data. The complex shear modulus (G*) was first calculated using the equation: $G^{*}=\sqrt{{G^{\prime }}^{2}+{G^{\prime \prime }}^{2}}$. Assuming isotropic material behavior, Young's modulus was then derived from the shear modulus using the standard relationship: E  =  2G(1 + ν) where G ≈ G′ (complex modulus from rheometer), ν = poisson's ratio (for soft materials like hydrogels, assume near 0.5). To assess the gelation speed, hydrogel was prepared by adding 0.2 mg mL^−1^ of Nile Red (Sigma) to achieve final concentrations of 10% and 16%. The mixture underwent 1 h of sonication and was loaded into a 31G needle syringe. Meanwhile, a vial containing 20 mL of water was heated to 37 °C, and the hydrogel was then injected to visually monitor gelation behavior. In parallel, rheological time‐sweep measurements at 37 °C were conducted for 50s to quantitatively assess gelation kinetics. For the in vivo hydrogel dissolution test, all animal experiments were approved by the IACUC at the Korea Institute of Science and Technology (KIST). 6‐week‐old female Balb/c mice (Orient Bio, South Korea) were anesthetized using 3% isoflurane in a mixture of oxygen and nitrogen. The hydrogel was mixed with 0.2 mg mL^−1^ nile red to achieve concentrations of 10% and 16% and sonicated for 1 h. Then, 50 µL of hydrogel samples were loaded into a 31G needle syringe and injected subcutaneously into the dorsal back skin of mice, with the left side receiving the 10% and the right side receiving the 16%. The retention of hydrogel was monitored at prearranged time points using an IVIS spectrum imaging system (Caliper, USA).

### Assessment of the Chemical Properties of I‐5 Hydrogel

Fourier Transform Infrared (FT‐IR) spectra of the hydrogels were recorded using a Nicolet iS20 spectrometer (Thermo). Samples were dissolved in chloroform, and spectra were collected in the range of ≈4000–600 cm^−1^ at a resolution of 4 cm^−1^ with 32 scans per sample. Background correction was automatically applied prior to each measurement. All measurements were conducted at room temperature.

### Cryogenic Scanning Electron Microscopy

The cryo‐SEM experiments were conducted using a Quanta 3D FEG microscope (FEI, Netherlands) with an Alto 2500 cryo‐transfer system (Gatan, UK). The I‐5 hydrogel was complexed with 1 × 10^6^ cultured NSCs at a concentration of 10 wt%, while cell‐free hydrogels at concentrations of 10%, 13%, and 16% were also prepared. The samples were rapidly frozen in liquid nitrogen and transferred to a pre‐evacuated freezing chamber at −190 °C, maintained at a pressure of 10^−5^ mbar. Metal deposition was achieved via sputtering with a 3‐mA current, and the samples were then transferred to a microscope chamber also pre‐evacuated to 10^−5^ mbar at −190 °C. Cryo‐SEM images were taken using a 5‐keV beam at 11.8 pA.

### Tissue Processing and Histological Experiments

Animals were deeply anesthetized using a mixture of ketamine (80 mg kg^−1^) and xylazine (10 mg kg^−1^), followed by intracardiac perfusion with cold PBS and 4% paraformaldehyde (PFA) in 0.1 M phosphate buffer. The tissue covering the lesion epicenter of the spinal cord (± 0.9 mm rostral and caudal) was carefully dissected out, and then post‐fixed with 4% paraformaldehyde for 6 h at 4 °C, followed by cryoprotection in a series of 10% and 30% sucrose solutions for 5 days. The spinal cord tissues were embedded in optimal cutting temperature compound and then sectioned longitudinally at a thickness of 20 µm using a CM1900 cryostat (Leica). To assess lesion cavities, tissue sections were immersed in a staining solution composed of 240 mL of 0.2% Eriochrome Cyanine RC (ECRC) and 10 mL of 10% FeCl3·6H2O in 3% HCl for 5 min, and then washed with running tap water for 5 min. Next, the tissues were treated with 1% NH_4_OH for differentiation and counterstained with eosin solution to make the lesion cavities induced by hydrogel injection more discernible. Picrosirius red staining (abcam, ab150681), which visualizes total collagen fibrils by binding to the aligned basic amino acids on the collagen molecule regardless of the type,^[^ ^32^ ^]^ was performed to evaluate the fibrotic environment within the hydrogel‐created ECM according to the manufacturer's instruction. For immunohistochemical staining, tissue sections were blocked in a 10% normal goat serum and 0.3% Triton‐X in PBS for 1 h at RT. Then, the primary antibodies were incubated in a blocking solution at 4 °C overnight. The following primary antibodies were used: chicken anti‐GFP (Abcam, ab13970, 1:3000), rabbit anti‐fibronectin (Sigma, F3648, 1:300), mouse anti‐fibronectin (Millipore, MAB1940, 1:100), rabbit anti‐GFAP (DAKO, Z0334, 1:2000), chicken anti‐GFAP (Abcam, ab4674, 1:5000), rabbit anti‐phospho p44/42 MAPK (ERK1/2, Cell signaling, 4370S, 1:200), rabbit anti‐Iba1 (Wako, 019–19741, 1:1000), rabbit anti‐5‐hydroxytryptamine (5‐HT, Immunostar, 20 080, 1:5000), mouse anti‐synaptotagmin (Millipore, MAB5200, 1:500), mouse anti‐nitrotyrosine (Abcam, clone HM.11, 1:300), and rabbit anti‐Tubb3 (Sigma, T2200, 1:2000). After washing off unbound primary antibodies with PBS three times for 5 min each, the samples were incubated with the appropriate fluorescent Alexa Fluor secondary antibodies (Thermo) for 2 h at RT. Following the antibody washings, tissue sections were mounted with an anti‐fade DAPI solution (Vector). Images were then obtained using an LSM 800 confocal laser‐scanning microscope (Carl Zeiss), an Axio Scan.Z1 slide scanner (Carl Zeiss), or an LSM 900 confocal laser‐scanning microscope with Airyscan super‐resolution (Carl Zeiss).

### Analyzing Images of Histological Samples

To evaluate the cell number of NSCs in vivo, three consecutive spinal cord tissues (300 µm interval between sections) covering the lesion epicenter were chosen for each animal. Images were acquired from the Zeiss Axio Scan.Z1 slide scanner (Carl Zeiss) microscope at a 200× magnification. The lesion epicenter was delineated by using GFAP immunoreactivity. Graft success was determined by the presence of distinguishable grafts within the lesion epicenter in at least one tissue section. The number of DAPI and GFP double positive cells was counted in each tissue section, and the average cell number per animal was calculated, as described in our previous study.^[^ ^47^ ^]^ To evaluate the expression of phosphorylated extracellular signaling‐related kinase 1/2 (phospho‐Erk1/2) in grafted neural stem cells (NSCs), images were captured using a confocal laser‐scanning microscope with a 20× objective lens and consistent exposure settings across all groups. Four regions of interest (ROIs) from three sections per animal were randomly placed within the lesion epicenter to cover the noticeable GFP positive engraftment. The number of NSCs doubly positive for GFP and phospho‐Erk1/2 was manually counted, and the percentage of the double positive cells out of the total GFP‐positive cells within ROIs was calculated. The percentage of NSCs that differentiated into either neurons or astrocytes was determined using the same method. To assess the expression of fibrotic matrix formation at varying hydrogel concentrations, images of fibronectin and Picrosirius red staining were obtained using Zeiss Axio Scan.Z1 slide scanner (Carl Zeiss) with 200× magnification. Two regions of interest (500 µm × 500 µm) were selected from the brightest areas within the lesion epicenter in each section, and four regions of interest per animal were analyzed to calculate an average value. To quantify the 5‐HT axonal ingrowth into the hydrogel‐created matrix, a single spinal cord section containing the largest NSC grafts was selected. The length of 5‐HT axons within hydrogel‐induced matrix was manually measured using the NeuronJ plug‐in in the ImageJ software. Co‐localization of doubly positive 5‐HT axons with NSC grafts was manually counted using the Zen Lite software (Carl Zeiss). To create a 3D reconstruction image visualizing synaptic contacts between host axons and grafted NSCs, we performed triple immunofluorescence staining using antibodies against GFP, 5‐HT, and synaptotagmin. Images were captured using a laser‐scanning microscope with a 63× objective lens and imported into IMARIS 9.0 software for 3D reconstruction. Background subtraction and the surface rendering method were used to create the 3D images following the instruction manual.

### NSC Adhesion and Spreading Assays on Substrates Covered with Hydrogels of Predefined Varying Stiffness

Cell culture vessels coated with polyacrylamide (PAA) hydrogel, featuring different elastic modulus levels, were purchased from Matrigen (http://matrigene.com). PAA hydrogel was bound to either coverslips in a 12‐well polystyrene plate (#SW12‐EC, Matrigen) or glass‐bottom coverslips in a confocal dish (#SV3520‐EC, Matrigen). Hydrogel coverslips were pre‐coated with poly‐D‐lysine (Sigma #P6407, 0.01 mg mL^−1^) for 18.5 h at 37 °C and gently washed with distilled water three times followed by drying for 1 h at RT. Cultured primary NSCs ubiquitously expressing GFP grown as neurospheres were collected into a 15 mL conical tube and mechanically dissociated into single cells followed by plating them onto the hydrogel coverslips in a DMEM + 3% FBS medium for 24 h. Subsequently, cold 4% PFA was used to fix the cells for 15 min at RT, and then washed three times with PBS followed by blocking steps for 1 h with 10% normal goat serum containing 0.3% triton‐X in PBS for immunostaining with following antibodies: chicken anti‐GFP (abcam, #ab13970; 1:2000), rabbit anti‐phospho‐ERK1/2 (Cell signaling, 4370S, 1:200). For pharmacological inhibition of mechanosensitive ion channels, cultured NSCs were treated with GsMTx4 (Piezo1 inhibitor, abcam, ab141871; IC50 5 µM), Pico145 (TRPC1 inhibitor, Medchem, HY‐101507; IC50 1.3 nM), and Amiloride hydrochloride (TRPP2 inhibitor, Medchem, HY‐B0285A; 2.6 µM) in a DMEM + 3% FBS medium for 24 h. Dosages for various drug treatments were determined based on the IC50 value of each reagent. For quantitative evaluation of NSC adhesion on hydrogel substrate with different stiffness, three regions of interest (ROIs) per coverslip were randomly placed, and images were obtained using an LSM 800 confocal laser‐scanning microscope at 100× magnification with identical exposure settings across all the experimental groups. The number of GFP+/DAPI+ cells was manually counted using Zen lite software, and the counts from all images were averaged to acquire one value per replicate. For the quantitative assessment of morphological changes in NSCs upon spreading onto the hydrogel substrate, we utilized the analysis methods outlined in previous publications^[^ ^75^ *^,^* ^76^ ^]^ with a minor modification. Images from five ROIs per coverslip were acquired at 200× magnification. The cell outlines in the images were manually traced using ImageJ. The area and perimeter were then obtained using the measurement function in ImageJ. The total spreading area and perimeter were divided by the number of cells to obtain the average spreading area and perimeter per cell. For visualization of filamentous actin, cells grown on PAA hydrogel were fixed with 4% PFA for 15 min at RT, followed by three washes with HBSS. Cells were then incubated with Alexa Fluor 594 Phalloidin (Thermo, #A12381) according to the manufacturer's instructions. Images were acquired using an LSM800 laser scanning confocal microscope (Carl Zeiss) equipped with a 40× objective. For each replicate, five randomly selected ROIs per coverslip were imaged under identical acquisition settings, ensuring comparable intensity measurement across all groups. One to two coverslips were used for each biological replicate. The average number of cells analyzed per replicate ranged from 15 to 50. Quantitative image analysis was performed using ImageJ software with a predefined threshold setting that was consistent across all groups.

### Live/Dead Cell Survival Assay In Vitro

Neurospheres were collected and dissociated into single NSCs and the cells were plated into Poly‐D‐Lysine pre‐coated hydrogel coverslips in a DMEM+3% FBS medium. NSCs were treated with or without sodium arsenite (NaAsO_2_, Merck, #106 277, 5 µM) to induce oxidative stress, mimicking the microenvironment in the injured spinal cord. After 24 h, cells were washed with HBSS and then incubated with the LIVE/DEAD Viability/Cytotoxicity Kit (Invitrogen, #L3224) according to the manufacturer's instructions. Briefly, cells were treated with 0.5 µM of calcein AM (495/517 nm) and 1 µM of ethidium homodimer‐1 (528/617 nm) for 20 min. Live cells convert nonfluorescent calcein AM into a green fluorescent calcein by hydrolyzing acetoxymethyl ester. Ehidium homodimer‐1 enters cells with damaged membranes, thereby producing a red fluorescence. Subsequently, images were captured from five randomly selected ROIs on a coverslip using a fluorescence microscope with 100× magnification. The images were then converted to 8‐bit and adjusted using identical thresholds with post‐processing using the Watershed function. The number of dead cells (red) was automatically quantified using the ImageJ software with an automatic cell counting module. To evaluate the potential damage to NSCs during an injection procedure, cell suspensions were loaded into a 26G Hamilton syringe (Hamilton #80 330) and then injected into a 24‐well culture plate using the same parameters used for in vivo injection (10 µL min^−1^, 1 min rest). After 30 min, the live/dead assay was conducted as described above using cells obtained before injection and those injected using a 26G Hamilton syringe. For live/dead cell survival assay in 3D hydrogel cultures, 1 × 10^6^ NSCs were resuspended in 70 µL of 10% or 16% hydrogel on ice and seeded into cell culture inserts (Falcon, #353 097). The hydrogel and NSC complexes were incubated at 37 °C for 30 min to allow sufficient gelation. Then, the inserts were transferred to a 24‐well culture plate (SPL, 13 485) containing the NSC culture medium (DMEM + 3% FBS). After 24 h, cells were stained with 0.5 µM calcein AM and 1 µM ethidium homodimer‐1 for 1 h at 37 °C, followed by 30 min at room temperature to permit sufficient dye penetration. The inserts were then placed on confocal imaging dishes (SPL, #101 350). Z‐stack images spanning a total depth of 495 µm were acquired at 5 µm intervals. Five images at different Z‐stack levels with 10‐µm intervals were selected from the Z‐stack images acquired at the center of each culture, with a field of view of 408 193.21 µm^2^. Live and dead cells were manually quantified using Zen Blue software (Ver. 2.6), and the survival rate of NSCs in 3D hydrogel culture was calculated as the percentage of dead cells (red) out of the total number of stained cells (both green and red). For the survival assay of NSCs subjected to electroporation of siRNA, cells were allowed to recover in the 2 mL of pre‐warmed culture medium at 37 °C incubator for 5 min before being embedded within the hydrogel.

### Calcium Imaging

For spontaneous intracellular Ca^2+^ imaging, glass bottom coverslips or hydrogel‐bound coverslips in a confocal dish (Matrigen, CA, #SV3520‐EC) were pre‐coated with Poly‐D‐Lysine for 18 h in a 37 °C incubator. Dissociated NSCs were plated and incubated for 24 h, and then the culture medium was washed out with HBSS. Subsequently, NSCs were loaded with 2 µM of membrane‐permeable calcium dye Fluo‐4 AM (Invitrogen, #F14201) in Hanks balanced salt solution (HBSS, Invitrogen) for 30 min in a 37 °C incubator, according to the manufacturer's instruction. The cells were washed three times with HBSS and then underwent complete de‐esterification of the dye for an additional 10 min at RT. Within 30 min of calcium dye loading, calcium imaging was performed in a bath solution containing 0.63 mM of calcium chloride, 0.245 mM of magnesium chloride, 0.2 mM of magnesium sulfate, 2.65 mM of potassium chloride, and 68.5 mM of sodium chloride using a LSM800 laser scanning microscope. Spontaneous Ca^2+^ oscillations were monitored using time‐lapsed images at 1‐s intervals for a duration of 1 min. For quantitative evaluation of Ca^2+^ oscillations, images were captured at 400× magnification. Five regions of interest (ROIs) were randomly selected, and 10 cells per image were analyzed, totaling 50 cells per group, using ImageJ with the Time Series Analyzer plugin. Three independent experiments were performed. Fluorescent signals were normalized with the baseline and presented as a ratio of the mean change in fluorescence (F_max_/F_0_). The baseline fluorescent signal (F_0_) was obtained from the first or last 10 s when the fluorescent level was the lowest. Calcium‐responsive cells were defined as cells showing more than a 20% increase over the baseline level, while cells showing less than 20% of the baseline were considered non‐responsive. For the drug pre‐treatment experiment, cells were treated with 20 µM of cytochalasin D (Sigma, #C8273) for 30 min prior to loading of Fluo‐4 AM. For the experiment with Yoda1 (Tocris, 5586, 100 µM), baseline Ca^2+^ activity was recorded for the first 30 s and applied the Yoda1 during the imaging.

### Electrophysiology

Whole‐cell patch‐clamp recordings were conducted at RT. The recording chamber was continuously perfused with a HEPES extracellular recording solution containing 135 mM NaCl, 5.4 mM KCl, 1.8 mM CaCl_2_, and 5 mM HEPES. The pH was adjusted to 7.4 with KOH and HCl, and the osmolality was set to 308 ± 2 mmol kg^−1^. Voltage‐clamp recordings utilized Cs^+^ solutions, with the Cs^+^ intracellular solution containing 90 mM Cs Methanesulfonate, 48.5 mM CsCl, 2 mM MgCl_2_, 5 mM Cs‐EGTA, 2 mM NaATP, and 5 mM HEPES. The pH of the Cs^+^ solution was adjusted to 7.2 with KOH, and the osmolality was set to 307 ± 2 mmol kg^−1^. Patch pipettes were pulled from borosilicate glass using a vertical micropipette puller (PC‐100, World Precision Instruments) and flame polished to achieve resistances of 5–7 MΩ. Signals were recorded using a Multiclamp 700B amplifier (Molecular Devices, Union City, CA), filtered at 2 kHz, and sampled at 10 kHz. A Picospritzer III delivered air pulses at 7 psi pressure to apply mechanical stimulation to the cells. The solution used in the pipette for mechanical stimulation had the same composition as the HEPES extracellular recording bath solution. Data were collected at a sampling frequency of 10 kHz and analyzed offline using Clampfit software. All group data are presented as mean ± SEM, and statistical comparisons were performed using a two‐way ANOVA. All drugs used in the electrophysiology experiments were purchased from Sigma‐Aldrich.

### Measurement of Elastic Modulus by Atomic Force Microscopy

Cellular elasticity was measured using atomic force microscopy (AFM) (Nano N8 Neos; Bruker Corporation, Germany) with force‐distance (FD) curve measurement in liquid condition.^[^ ^77^ ^]^ The FD curve was measured by using an AFM probe (Cont GD, Budget Sensors Inc, Bulgaria) composed of an Au‐coated cantilever and Si‐based conical tip. Detailed dimensions of the probe were as follows: resonance frequency, 13 kHz (± 4 kHz); force constant, 0.2 N m^−1^ (0.07–0.4 N m^−1^); cantilever length, 450 µm (± 10 µm); cantilever width, 50 µm (± 5 µm); cantilever thickness, 2 µm (± 1 µm); tip height, 17 µm (± 2 µm); tip radius < 10 nm. The half‐cone angle of the conical tip along the cantilever axis ranged from ≈20 to 22.5°. The load force was set at ≤ 10 nN to minimize damage to the cell membrane, and the loading rate of the probe was ≈1 µm s^−1^. FD curve measurement was performed at 10 points per cell for 20 to 31 cells of each condition. The cellular elasticity was measured around the nucleus, where the cell thickness was ≈1 µm or more, to avoid stiffness effects due to the nucleus and matrix. NSCs were seeded on the hydrogel‐bound coverslips and incubated for 24 h in DMEM + 3% FBS and then the cells fixed with 3.7% paraformaldehyde for 15 min at RT followed by washing three times with PBS. The samples were submerged in PBS at 4 °C until AFM measurement.

### RNA Extraction and qPCR

Total RNA was isolated from dissected tissues or cultured cells using Trizol (Invitrogen) according to the manufacturer's instructions. RNA quantity and purify were analyzed using a NanoDrop Life Spectrophotometer (Thermo Fisher Scientific) at 260 nm. One microgram of RNA was reverse transcribed to cDNA using the manufacturer's protocol (Cellsafe, #CDS‐100). One microliter of cDNA was added to SYBR green Master Mix (Takara) containing 10 pM of the primer pairs listed in the Table S3, Supporting Information. Quantitative real‐time PCR was performed using a 7500 Real‐Time PCR system (Applied Biosystems, Foster City, CA, USA) according to the manufacturer's protocol. The cycling conditions were 95 °C for 30 s and ≈54.8–61.5 °C for 5 s for a total of 40 cycles. Melting curves were generated after the final extension step, and Ct values were calculated using the Applied Biosystems 7500 software. The Ct values of the target genes were normalized to the 18S rRNA housekeeping gene as an internal control.

### Gene Silencing Experiment with siRNA

Piezo loss of function experiments were performed using small interfering RNA (siRNA, Dharmacon, #361 430). Briefly, five million dissociated NSCs were transferred into a nucleofector cuvette containing Nucleofector solution (Lonza, Rat Neural Stem Cell Nucleofector Kit, #VPG‐1005) with 2 µM of siRNA against Piezo1 or scrambled siRNA. Subsequently, the cuvettes were inserted into a Nucleofector 2b (Lonza) using Rat Neural Stem Cell Nucleofector Kit (Cat No: VPG‐1005) followed by electroporation. The cells were then allowed to recover for 24 h in a culture plate before the subsequent experiments.

### CRISPR/Cas9‐Mediated Piezo1 Editing in NSCs

The CDS region of rat *Piezo1* gene was screened for sgRNA using the Cas‐Designer tool. (http://www.rgenome.net/).^[^ ^78^ ^]^ Small guide RNAs (sgRNAs) were selected based on filtering with mismatch criteria of 1,0,0. These sgRNAs were synthesized in vitro and transfected into C6 gloma cells (ATCC, CCL‐107) for screening. After transfection, lead sgRNA was selected based on their high gene editing activity, as determined by targeted deep sequencing, and by the downregulation of target gene expression at the mRNA level. For targeted gene editing in NSCs, sgRNA and Cas9 protein were introduced via electroporation using a 4D‐Nucleofector (Lonza, P3 Primary Cell 4DNucleofector X Kit) as a ribonucleoprotein (RNP) complex. Briefly, the RNP complex was formed by mixing 1 µg Cas9 protein with 4 µg sgRNA and incubating at room temperature for 10 min. This complex was then electroporated into 4 × 10^5^ rat NSCs using 20 µL of primary P3 buffer (Lonza). At least 72 h after transfection, genomic DNAs (gDNAs) were extracted using a genomic DNA extraction Kit (Favorgen) according to the manufacturer's protocol. To identify gene editing efficiency and the exact sequence edited, targeted deep sequencing was performed using gDNAs as previously described.^[^ ^79^ ^]^ Briefly, gDNAs were amplified using specific primer sets. Paired‐end sequencing was then performed using an Illumina MiSeq (Primer used in this study are described in Table S3, Supporting Information). Indel frequency and sequence edits were analyzed using the online Cas‐Analyzer tool (www.rgenome.net).

### Statistical Analysis

Statistical analyses were performed using GraphPad Prism software (version 8.0.2). Unpaired Student's *t*‐test (two‐tailed) was used to compare the mean values of two groups. One‐way ANOVA followed by Tukey's *post hoc* analysis was used for the mean values of three or more groups. Two‐way ANOVA followed by Bonferroni's *post hoc* analysis was performed in experiments with two independent variables.

## Conflict of Interest

SCS is the CEO of Nexgel Biotech, a company founded to commercialize the results of his research on hydrogel. BGK is a scientific consultant for Nexgel Biotech.

## Author Contributions

These two authors H.H.P. and Y.K. equally contributed to this work. Conceptualization: H.H.P., D.H.H., Y.M.K., S.C.S., and B.G.K. Methodology: H.H.P., K.S.K., J.Y.L., J.M.P., Y.M.K., and B.G.K. Investigation: H.H.P., Y.K., B.S.J., S.G., D.H.H., Y.S., S.A.J., H.G.S., H.J.K., S.L., S.K., and K.I.L. Visualization: H.H.P., Y.K., B.S.J., S.A.J., and H.S.K. Supervision: K.S.K., J.Y.L., J.M.P., Y.M.K., S.C.S., and B.G.K. Writing‐original draft: H.H.P., Y.K., A.E., H.J.K., and B.G.K. Writing – review & editing: J.Y.L., J.M.P., Y.M.K., and B.G.K.

## Supporting information



Supporting Information

Supplemental Movie 1

Supplemental Movie 2

Supplemental Movie 3

Supplemental Movie 4

Supplemental Movie 5

## Data Availability

The data that support the findings of this study are available from the corresponding author upon reasonable request.
