# Supplementary material for: Mechanical Environment Afforded by Engineered Hydrogel Critically Regulates Survival of Neural Stem Cells Transplanted in the Injured Spinal Cord via Piezo1‐Mediated Mechanotransduction
Source: Adv Sci (Weinh). 2025 Nov 3;13(3):e07160. doi: 10.1002/advs.202507160 (PMC12806497; doi:10.1002/advs.202507160)
Supplement: Supplementary file 1 — Supporting Information [file ADVS-13-e07160-s006.docx]

Supplementary Materials for

**Mechanical environment afforded by engineered polymer hydrogel critically regulates survival of neural stem cells transplanted in the injured spinal cord via Piezo1-mediated mechanotransduction**

Hee Hwan Park^1,2†^, Yurim Kim^4,5†^, Byeong Seong Jang^1,2^, Simay Genişcan^1,2^, Dong Hoon Hwang^1,11^, Yeojin Seo^1,2^, Seung-Ah Jee^1,2^, Hyo Gyeong Seo^1,2^, Hyung Soon Kim^1^, Ariandokht Einisadr^1,2^, Ho-Jeong Kim^5,6,^, Seolhee Lee^5,6,^, Sangwoo Kwon^8^, Kyung Sook Kim^8^, Kang In Lee^9^, Jae Young Lee^9,10^, Joo Min Park^5,6,7^, Young-Min Kim^4,5^*, Soo-Chang Song^4,5^*, Byung Gon Kim^1,2,3^*

1 Ajou University School of Medicine, Department of Brain Science, Suwon, 16499, Republic of Korea.

2 Neuroscience Graduate Program, Department of Biomedical Sciences, Ajou University Graduate School of Medicine, Suwon, 16499, Republic of Korea.

3 Ajou University School of Medicine, Department of Neurology, Suwon, 16499, Republic of Korea.

4 Center for Biomaterials, Korea Institute of Science and Technology, Seoul, 02792, Republic of Korea

5 Center for Cognition and Sociality, Institute for Basic Science, Daejeon, Republic of Korea.

6 Department of Biomedical Engineering, Ulsan National Institute of Science and Technology (UNIST), Ulsan, Republic of Korea.

7 University of Science and Technology (UST), Daejeon, Republic of Korea

8 Department of Biomedical Engineering, College of Medicine, Kyung Hee University, Seoul 130-710, Republic of Korea.

9ToolGen Inc., Seoul, 07789, Republic of Korea

^10^Ajou University School of Medicine, Department of Anatomy, Suwon. 16499, Republic of Korea

^11^ Present address: Wonju Medical Industry Technovalley, Wonju 26354, Republic of Korea

† These two authors (HHP and YK) equally contributed to this work.

* To whom correspondence should be addressed;

Young-Min Kim davidkim@kist.re.kr

Soo-Chang Song [scsong@kist.re.kr](mailto:scsong@kist.re.kr)

Byung Gon Kim kimbg@ajou.ac.kr

**Figure S1. Syringe needle pressure does not influence the NSC viability**

**(A)** Schematic diagram of the cell viability test caused by syringe needle pressure during injection. **(B)** Representative images of cell survival assay before and after injection. Neurospheres were enzymatically dissociated into single cells, loaded into a Hamilton syringe, and injected into culture medium, mimicking the *in vivo* transplantation study. Live and dead cells were stained using LIVE/DEAD™ Viability/Cytotoxicity Kit. Live cells labeled with Calcein-AM (green) and dead cells labeled with Ethidium Homodimer-1 (red). Scale bar = 100 μm. **(C)** Quantitative graph showing the percentage of dead cells before and after needle injection. Each dot represents one experiment, with averaging of three injection attempts per experiment. Four independent experiments were conducted. Error bars represent SEM.

**Figure S2. Combination of a growth factor or ECM protein does not improve the survival of NSCs transplanted with hydrogel
(A)** Representative images of longitudinal spinal cord sections obtained from animals transplanted with NSCs in 10% hydrogel combined with insulin growth factor-1 (IGF-1) or laminin. The samples were collected at 4 weeks post-transplantation. Spinal cord sections were visualized using eriochrome cyanine and eosin staining (left panel). GFP indicates surviving NSC grafts (green) and GFAP demarcates the lesion areas (magenta, middle panel). Boxed regions are magnified on the right side. Scale bar = 1000 μm.


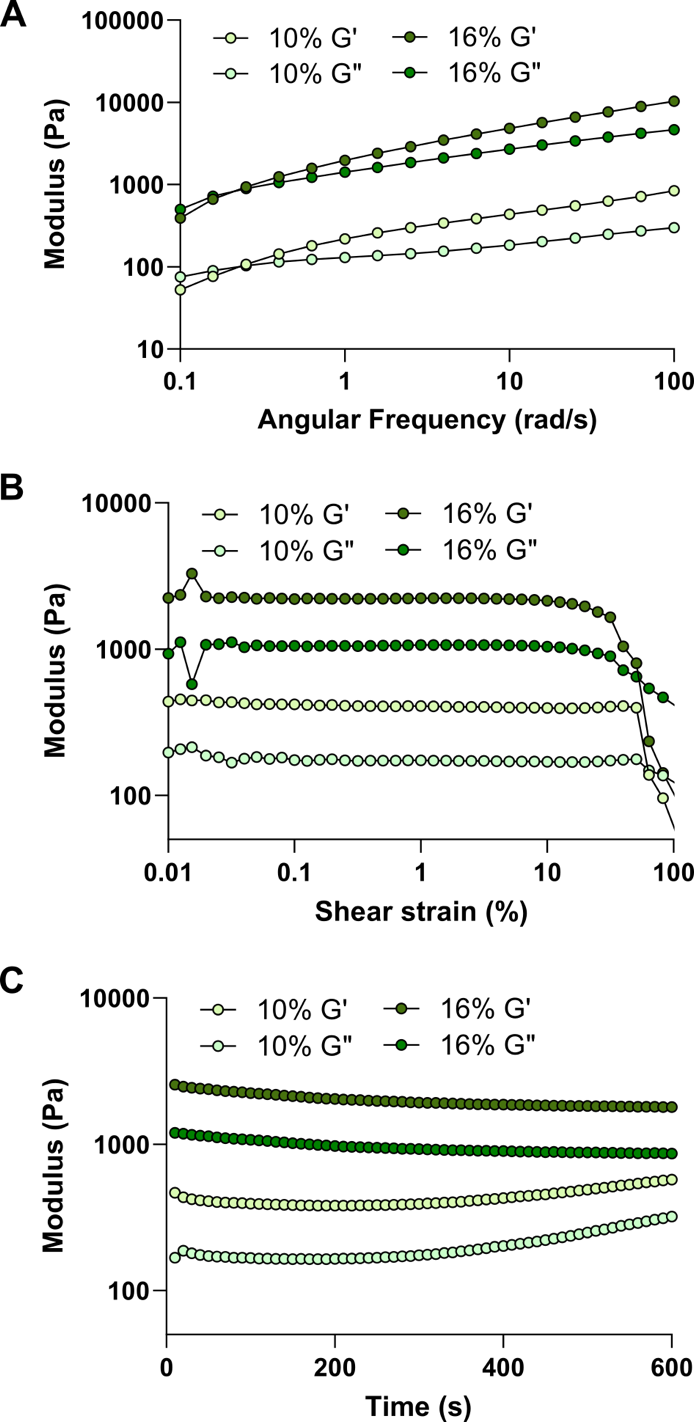


**Figure S3. Rheological analysis of 10 % and 16 % I-5 hydrogel.**

**(A**) Frequency sweep (0.1–100 rad/s) showing storage (G′) and loss (G″) moduli. **(B)** Strain sweep (0.01–100 %) demonstrating the linear viscoelastic region and yield point. **(C)** Time sweep (0–600 s) at 37 °C assessing modulus stability over time.

**Figure S4. No difference in the extent of inflammatory reactions between 10% and 16% hydrogel groups**

**(A-B)** Representative images of longitudinal spinal cord sections obtained from animals that received only NSCs or those complexed with 10% or 16% hydrogel. The samples were collected at 4 weeks post-transplantation. Neuroinflammation was evaluated by Ionized calcium-binding adaptor molecule 1 (Iba1) immunostaining. Boxed regions correspond to magnified views below (B1-B3). Asterisks indicate cystic cavity spaces. Scale bars = 500 μm.

**Figure S5. A difference in hydrogel concentration does not influence the extent of fibrotic matrix formation**

**(A)** Representative images of longitudinal spinal cord sections showing fibronectin staining (top panel) and picrosirius staining for total collagen matrix (bottom panel). No significant differences in fibrotic extracellular matrix formation were observed between groups. **(B)** Quantitative graphs comparing two groups. N = 5 and 7 for the 10% and 16% hydrogel groups. Error bars represent SEM. Scale bars = 500 μm.

**
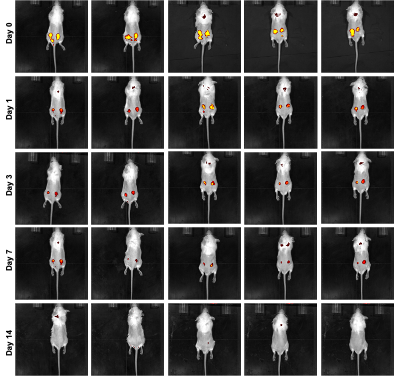
**

**Figure S6. Hydrogel degradation kinetics with different concentrations of hydrogel**

Representative IVIS spectrum imaging system (Caliper, USA) images showing the mass loss of 50 µl of Nile Red-labeled hydrogel over 0, 1, 3, 7, and 14 days following hydrogel injection. A total of 50 µl of hydrogel samples were loaded into a 31G needle syringe and injected subcutaneously into the dorsal back skin of six-week-old Balb/c mice, with the left side receiving the 10% and the right side receiving the 16%. The left and right panels correspond to 10% and 16% hydrogels in the same animal subject. A total of five animals were monitored during the experimental period.


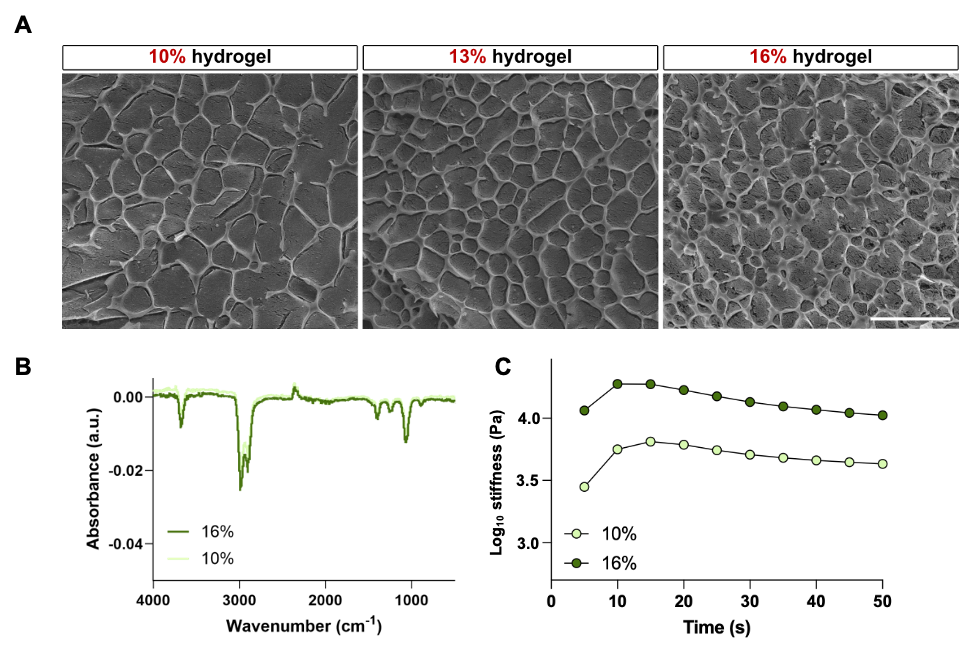


**Figure S7. Stiffness as an independent parameter modulating neural stem cell (NSC) behavior in I-5 hydrogels.**

**(A)** Cryo-SEM images of 10 %, 13 %, and 16 % I-5 hydrogels, showing internal porous structures. Scale bars = 5 µm. **(B)** FTIR spectra of 10 % and 16 % of I-5 hydrogels measured in the range of 4000-750 cm^-1^. **(C)** Time-dependent viscosity profiles of 10 % and 16 % I-5 hydrogels over 150 s at 37 °C.

**Figure S8. Measurement of the hydrogel substrate stiffness using atomic force microscopy (AFM)**

**(A)** A schematic diagram illustrating the experimental setup of the AFM to measure the elastic modulus of the NSC plasma membrane. **(B)** Quantitative graph measuring the elastic modulus of the hydrogel culture substrates with varying degrees of mechanical stiffness. N = 12, 25, and 5 cells for 100 kPa, 25 kPa, and 12 kPa groups, respectively. *** indicate *p* < 0.001 compared to the 100 kPa group, and ### indicate *p* < 0.001 compared to the 25 kPa group by one-way ANOVA followed by Tukey’s *post hoc* analysis. Error bars represent SEM.

**Figure S9. Cytochalasin D treatment destabilizes filamentous actin cytoskeleton in NSCs in a dose-dependent manner**

Representative images of GFP-positive NSCs cultured on 25 kPa hydrogel substrate and treated with 2 or 20 μM cytochalasin D (actin destabilizer) for 30 min before fixation. Actin filaments (F-actin) were visualized using Alexa Fluor 594-Phallodin. Scale bars = 5 μm.

**Figure S10. Neither TRPC1 nor TRPP2 channels influence NSC adhesion on a rigid hydrogel substrate.**

**(A)** Representative images of NSCs cultured on the 25 kPa hydrogel substrate treated with TRPP2 inhibitor (amiloride hydrochloride) for 24 h. **(B-D)** Quantitative graphs comparing the number of cells adhered **(B)**, areas of cell spreading **(C)**, and the perimeter of cell boundary **(D)**. Each dot represents an independent culture replicate, with each replicate being a measurement from one coverslip. **(E)** Representative images of NSCs cultured on 25 kPa hydrogel substrate treated with TRPC1 inhibitor (Pico145) for 24 hrs. **(F-H)** Quantitative graphs comparing the number of cells adhered **(F)**, areas of cell spreading **(G)**, and the perimeter of cell boundary **(H)**. Each dot represents an independent culture replicate, with each replicate being a measurement from one coverslip. Error bars represent SEM. Scale bars = 20 μm.

**Figure S11. Representative raw current traces from NSCs in response to pressure stimulation**

Raw current traces were obtained under a voltage clamp mode at varying holding voltages. Short air pulses were applied to NSCs, lasting 10 ms, at a pressure of 7 psi using a Picospritzer device.

**Figure S12. Validation of Piezo1 knockdown in NSCs**

**(A**) Quantitative PCR (qPCR) analysis of *Piezo1* mRNA expression in NSCs cultured on substrates of varying stiffness. N = 3 independent cultures. **(B)** Validation of Piezo1 knockdown in NSCs by electroporation. *** indicates *p* < 0.001 by one-way ANOVA followed by Tukey’s *post hoc* analysis. N = 3 independent cultures. Error bars represent SEM.

**Figure S13.  Screening of CRISPR/Cas9 associated gRNA sequences targeting rat Piezo1**

**(A**) Targeted deep sequencing-based gene editing efficiency of 13 candidate sgRNA sequences in C6 glioma cells. Arrows indicate sequences with Indel frequency higher than 90%. **(B)** Quantitative RT-PCR measuring the levels of Piezo1 in C6 glioma cells that were edited using 6 sgRNA sequences that showed the highest Indel efficiency. An arrow indicates the sequence showing the largest reduction of Piezo1 mRNA level. Error bars represent SEM.

**Figure S14. Validation of functional Piezo1 knockdown by Crispr/Cas9 system**

**(A)** Representative time-lapse images of intracellular calcium uptake in NSCs cultured on 0.2 kPa hydrogel substrate. Piezo1 agonist Yoda-1 was added to the culture plate at 100 μM after 30 sec baseline recording. Calcium uptake was visualized using Fluo4-AM. Scale bars indicate 20 μm. **(B-C)** Quantitative graphs comparing the intracellular calcium levels in response to Piezo1 agonist stimulation. *** indicate p < 0.001 comparing Cas9 only with Cas9 + sgPiezo1, and # indicates p < 0.05 comparing Cas9 + sgScr with Cas9 + sgPiezo1 (B). * indicates p < 0.05 (C) by one-way ANOVA followed by Tukey’s *post hoc* analysis. N = 4 biological replicates (40 cells per replicate), derived from two independent cultures.
